# Iron and Nickel Substituted Perovskite Cobaltites for Sustainable Oxygen Evolving Anodes in Alkaline Environment

**DOI:** 10.1002/cssc.202401403

**Published:** 2024-11-04

**Authors:** Henrik Petlund, Alaa Faid, Junjie Zhu, Anuj Pokle, Truls Norby, Svein Sunde, Athanasios Chatzitakis

**Affiliations:** ^1^ Department of Chemistry, Centre for Materials Science and Nanotechnology University of Oslo Gaustadalléen 21, NO-0349 Oslo, Norway; ^2^ Department of Materials Science and Engineering Norwegian University of Science and Technology (NTNU) NO-7491 Trondheim Norway; ^3^ Institute for Energy Technology (IFE), NO-2007 Kjeller Norway; ^4^ Department of Physics, Centre for Materials Science and Nanotechnology University of Oslo POB 1048, NO-0316 Oslo Norway

**Keywords:** Alkaline water electrolysis, Perovskites, Earth abundant catalysts, Oxygen evolution reaction, In-situ Raman spectroscopy

## Abstract

Perovskite oxides have great flexibility in their elemental composition, which is accompanied by large adjustability in their electronic properties. Herein, we synthesized twelve perovskite oxide‐based catalysts for the oxygen evolution reaction (OER) in alkaline media. The catalysts are based on the parent oxide perovskite Ba_0.5_Gd_0.8_La_0.7_Co_2_O_6‐δ_ (BGLC587) and are synthesized through the sol‐gel citrate synthesis route. To reduce the demand on cobalt (Co), but also increase the intrinsic catalytic activity of BGLC587 for the OER, we substitute Co on the B‐site with certain amounts of Fe and Ni, synthesizing catalysts of the general formula Ba_0.5_Gd_0.8_La_0.7_Co_2‐x‐y_Fe_x_Ni_y_O_6‐δ_. A plethora of physicochemical and electrochemical methods suggest that an Fe content between 30 % and 70 % increases the intrinsic catalytic activity of BGLC587, while Tafel slopes in combination with in‐situ Raman spectroscopy suggest the rate determining step is likely a proton‐exchange reaction, progressing possibly through the lattice oxygen mechanism (LOM). We apply one of the optimized, Co‐substituted perovskites in a monolithic, photovoltaic (PV)‐driven electrolysis cell and we achieve an initial solar‐to‐hydrogen (STH) conversion efficiency of 10.5 % under one sun solar simulated illumination.

## Graphical abstract

Perovskite oxide catalysts with reduced Co content, substituted by Fe and Ni, are synthesized and studied for their activity in the oxygen evolution reaction in alkaline media. An increase in the intrinsic catalytic activity (obtained by the (*R*_ct_ *C*_dl_)^−1^ product) of the base material (Ba_0.5_Gd_0.8_La_0.7_Co_2_O_6‐δ_) is seen for an as high as 70 % substitution of Co by Fe. Application in a monolithic PV‐driven electrolysis cell reaches a 10.5 % STH efficiency.

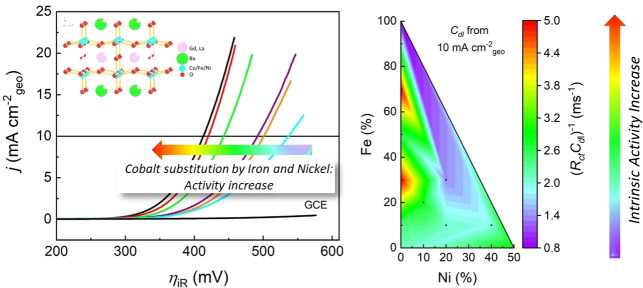

## Introduction

The oxygen evolution reaction (OER) is a very important reaction, which is complementary to H_2_ evolution but also to CO_2_ reduction. The OER is a four‐electron process that involves several intermediate steps and most OER catalysts (in both acidic and alkaline conditions) show slow reaction kinetics. This is widely recognized and the research on improving catalyst materials for it is immense.^[1]^ In acidic conditions, Ir‐based catalysts are usually employed due to their high stability, but the high cost and scarcity of Ir, makes its large scale deployment for the OER impossible. In alkaline conditions, Ni‐based catalysts have shown good electrocatalytic activity and together with their relatively high abundance have been seen as promising OER catalysts for scaled‐up applications. Moreover, their modification with Fe has led to further improvement in their catalytic activity and have been extensively studied for the OER in alkaline media.^[2]^

Lately, perovskite‐based oxides have also shown great promise in catalyzing the OER in alkaline conditions.^[5]^ The main advantages of perovskite‐based catalysts are their high flexibility in elemental composition, highly‐tolerant crystal structures and high adjustability in their electronic properties. In many instances, they have been used as model systems to study fundamental phenomena, linking for example catalytic activity with structural changes.^[8]^ We have previously studied a class of perovskite oxides based on the general formula Ba_1−x_Gd_0.8‐y_La_0.2+x+y_Co_2_O_6‐δ_ (BGLC), that showed high intrinsic catalytic activity and stability as anodes in alkaline media.[^9^, ^11^] These catalysts are primarily developed for high temperature solid oxide electrolysis cells, achieving record breaking steam electrolysis efficiencies,^[13]^ therefore holding great promise as universal catalysts for both low and high temperature water splitting applications.

To improve their performance in the low temperature regime, but at the same time minimize the use of cobalt (Co), which is linked to severe societal and environmental impacts,^[14]^ the aim of this work is to substitute Co with more benign elements, such as iron (Fe) and nickel (Ni). As outlined above, both Ni‐ and Fe‐based electrocatalysts have showed good activity and stability for the OER in alkaline media,^[17]^ while Ba_0.5_Sr_0.5_Co_0.8_Fe_0.2_O_3‐δ_ (BSCF82) is currently the benchmark perovskite oxide electrocatalyst for the OER,^[8]^ we hypothesized that Ni‐ and Fe‐substitution of BGLC should improve its OER performance. As an example, Fe substitution in PrBa_0.8_Ca_0.2_Co_2_O_6‐δ_ (PBCO) resulted in a higher O content, where δ decreased from 0.25 to 0.03.^[19]^ Since PBCO is acceptor‐doped, the higher oxygen content because of Fe‐doping will lead to an increase in the concentration of electron holes as charge compensating defects.

Herein, we have synthesized a series of OER electrocatalysts based on the general formula, Ba_0.5_Gd_0.8_La_0.7_Co_2‐x‐y_Fe_x_Ni_y_O_6‐δ_ (BGLCFN), while having Ba_0.5_Gd_0.8_La_0.7_Co_2_O_6‐δ_ (BGLC587) as the base material.^[11]^ After an initial screening, the most active Co‐substituted perovskites have been further characterized by physicochemical, electrochemical and in‐situ spectroscopic techniques. We show through intrinsic electrocatalytic activity measurements that Co substitution by Fe and Ni leads to increased OER activity, while in‐situ Raman spectroscopy was crucial to deduce the rate determining step and elucidate a possible reaction pathway for the evolution of oxygen gas.

## Experimental

### Catalyst Synthesis

All electrocatalysts were prepared by the sol‐gel citrate method, which typically included the following steps. First, citric acid (≥99.0 % Sigma‐Aldrich) was dissolved in de‐ionized (DI) water in a beaker on a hotplate with constant magnetic stirring. Second, using a ratio of 1 : 1 citric acid and moles of cations, a stoichiometric amount of precursors (BaCO_3_ 99.8 % Alfa Aesar, Gd(NO_3_)_3_ ⋅ 6H_2_O 99.9 % Sigma‐Aldrich, La(NO_3_)_3_ ⋅ 6H_2_O 99.99 % Sigma‐Aldrich, Co(NO_3_)_2_ ⋅ 6H_2_O ≥98 % Sigma‐Aldrich, Fe(NO_3_)_3_ ⋅ 9H_2_O ≥98 % Sigma‐Aldrich and Ni(NO_3_)_2_ ⋅ 6H_2_O ≥98 % Sigma‐Aldrich), containing the desired cations, were slowly added until they were fully dissolved. Third, the pH was adjusted dropwise with concentrated ammonia until reaching a pH value of 6–7 and until obtaining a clear solution.

Water was then evaporated from the solution on the hotplate at 150 °C until a viscous and eventually solid gel was formed. At this point, the beaker was covered with a watch glass and placed in a ventilated heating cabinet to obtain a more homogeneous temperature distribution. The beaker was left in the heating cabinet for 2 h at 250 °C to complete the combustion reaction. The ash obtained was crushed thoroughly in a mortar inside a fume hood and transferred to a magnesia crucible. The powder was fired at 450 °C for 2 h with a ramp rate of 300 °C h^−1^ to burn off most organic compounds. Then, the powder was crushed thoroughly a second time and calcined at 1100 °C for 8 h with a ramp rate of 250 °C h^−1^ to form the final powder. A list with the compositions of the electrocatalysts synthesized in this work, along with their respective abbreviations and nominal B‐site compositions are given in Table S1 in the supporting information (SI).

### Catalyst Ink Preparation and Electrochemical Characterization

Catalyst inks were prepared according to previous works,[^11^, ^20^] by dispersing 10 mg of catalyst powder, which had earlier been sieved in a vibratory sieve from Retsch using a 20 μm sieve, in a solution of 1 mL ethanol and 100 μL 5 wt.% Nafion®. The ink was ultrasonicated in an ice bath for 1 h prior to use.

All electrochemical measurements were carried out in a 1.0 M KOH electrolyte solution at room temperature (20±2 °C) and a Gamry REF3000 potentiostat was used to control the electrochemical experiments. Unless otherwise stated, all measurements were done using a three‐electrode setup with a graphite rod counter electrode (CE) and a saturated calomel electrode (SCE) reference electrode (RE). For long‐term stability measurements the SCE was replaced by a Hg/HgO (20 % KOH) reference electrode, which is the preferred reference electrode in alkaline solutions. Nevertheless, the SCE was always checked for drifts in potential prior to and after each set of electrochemical measurements. As working electrode (WE), both a rotating disk electrode (RDE), rotating at 1600 rpm, equipped with a glassy carbon electrode (GCE) tip (0.196 cm^2^) and C‐paper‐based (Toray TGP−H‐060) electrodes were used, depending on the measurement type. The electrolyte solution was saturated with O_2_ gas before and during all OER measurements. All overpotentials presented in this work were extracted at a current density of 10 mA cm^−2^ and calculated relative to the reversible potential of the OER: $E_{O_{2}/H_{2}O}^{0}$ =1.229 V vs RHE.

The preparation of the WE was done as follows. First, the GCE tip was polished to a mirror finish with alumina slurry (0.05 μm grains) on a polishing pad. Second, the tip was ultrasonicated in 1.0 M KOH for about 5 min and rinsed in DI water before left to dry in ambient air. This method was adapted from work by Wei, et al. and Faid, et al.^[21]^ Third, the electrocatalyst powder was loaded on the electrode by drop‐casting 6 μL of catalyst ink, corresponding to a catalyst loading of 0.28 mg cm^−2^. To prevent any bubbles of air sticking to the electrode surface upon immersing it in the electrolyte, the WE was covered with a 30 μL droplet of DI water.

Before electrochemical characterization, the GCE with as deposited catalyst was cleaned electrochemically by performing cyclic voltammetry (CV) from 1.0 to 0.03 V vs. RHE at 50 mV s^−1^ until steady‐state conditions were obtained, as suggested by Wei et. al.^[21]^ Subsequently, the catalyst was activated by CV at a scan rate of 10 mV s^−1^ from the non‐Faradaic region to the potential required to reach a current density of 10 mA cm^−2^. After 10 cycles of activation, most catalyst materials showed a steady‐state behavior and a linear sweep voltammetry (LSV) curve was recorded at a scan rate of 10 mV s^−1^.

To assess the electrochemical surface area (ECSA) through the double layer capacitance (*C*_dl_), CV was applied in the non‐Faradaic region according to the procedure in Supplementary Note 1 and Figure S13. Thereafter, electrochemical impedance spectroscopy (EIS) was carried out in the non‐Faradaic region, at the onset potential and the potential corresponding to 10 mA cm^−2^ to complement on the ECSA from CV and find the intrinsic catalytic activity (ICA) through the *R*_ct_ *C*_dl_ product.^[23]^ The details of the EIS procedure can be found in Supplementary Note 2 and Figure S14. Lastly, stepped chronopotentiometry (CP) was performed to derive Tafel slopes at truly steady‐state conditions and the steps of this method can be found in Supplementary Note 3 and Figure S15.

### Reference Electrode Calibration

Since pH values above 13 can be hard to determine precisely and they can also affect the measured potential with up to tens of mV,^[21]^ the potential of our SCE reference electrode vs. RHE was found from Equation 1,

(1) $$E_{RHE}=E_{measured}-E_{offset}$$

where *E_measured_* is the measured potential vs. the reference electrode of choice (here SCE)*. E_offset_* was found by CV measurements in the hydrogen evolution/oxidation reaction (HER/HOR) region with a polished Pt wire as the WE and a Pt mesh as the CE in H_2_ saturated electrolytes. The value of *E_offset_* was then determined from the mean value of the intercepts with the potential axis. The CV was done with constant magnetic stirring and at a scan rate of 1–2 mV s^−1^ to obtain close to steady‐state conditions. Prior to calibration, the cell was deaerated with Ar gas whereupon all connections were sealed with O‐rings, Si‐grease and Parafilm®. This method (adapted from Wei et al. and Niu et al.)^[21, 25],^ and the calibration results are presented in Supplementary Note 4 and Figure S16 in the SI.

### Faradaic Efficiency Measurements

The faradaic efficiency (FE) of the electrocatalysts was determined by measuring the amount of O_2_ and H_2_ gases evolved as a function of time with a 3000 Micro Gas Chromatograph (GC) from Agilent Technologies, which was calibrated by known O_2_ and H_2_ partial pressures. A C‐paper electrode with 5 mg cm^−2^ loading of catalyst material was used as WE with a Pt‐mesh RE/CE. More details for the preparation of the C‐paper‐based WE electrode for long‐term stability measurements can be found in Supplementary Note 5 and Figure S17 in the SI. A Pt foil was used as the CE and the FE measurements took place in a two‐electrode electrochemical cell equipped with a separator between the CE (cathode) and WE (anode) electrodes. Before measuring, the cell was purged free of O_2_ and N_2_ with Ar gas (Nippon Gases, 5.0).

### Physicochemical Characterizations

The structure and phase purity of pristine and post operation electrocatalysts were determined with powder X‐ray diffraction (PXRD), performed in a Bruker AXS D8 Discover with Bragg‐Brentano geometry using Cu−Kα1 (λ=1.54060 Å) and Cu−Kα2 (λ=1.54439 Å) radiation. The samples were measured between 10° and 70° 2θ with a step size of 0.02°. The analysis was performed in DIFFRAC.EVA V4.3 utilizing data from the Crystallographic Open Database (COD). The phase composition of the B‐site substituted electrocatalysts was found with Rietveld refinement in Topas V6 (Bruker) using cif‐files from previous work by Zhu et al.^[11]^ and the COD.

The surface morphology and phase composition of the electrocatalyst powders were studied in a Hitachi SU8200 scanning electron microscope (SEM) with acceleration voltages of 2–15 kV. A secondary electron (SE) detector was used for sample surface morphology and microstructural investigation, whereas a high‐angle backscattered electron, HA(T), detector was used to obtain mainly atomic number contrast (Z‐contrast). The elemental composition of the electrocatalysts was examined with Energy Dispersive X‐Ray Spectroscopy (EDS) using a Bruker XFlash 6–10 detector.

Selected compositions were further investigated with high‐resolution scanning transmission electron microscopy (STEM) and EDS (Super‐X detector) using a Thermo Fisher Scientific Cs‐corrected Titan G2 60–300 kV microscope operated at 300 kV. The bright field (BF) STEM images were recorded using a probe convergence semi‐angle of 23 mrad and collection semi‐angle of 0 to 22 mrad.

Surface area estimation of selected electrocatalysts was conducted by applying Brunauer‐Emmett‐Teller (BET) theory on adsoption isotherms acquired with a Belsorp Mini X (MicrotracBEL Corp.). The powders were activated in vacuum at 300 °C for about 3 h to remove any physisorbed water and hydrocarbons at the surface. Approximately 1 g of powder was used in each measurement and N_2_ was used as the adsorptive gas.

### In‐Situ Raman Spectroscopy

In‐situ Raman spectroscopy analysis was performed in a WITec alpha 300 R Confocal Raman spectrometer equipped with a 532 nm laser with a power of 20 mW. The Raman spectrometer was coupled with a Zeiss EC Epiplan 10× objective and G1: 600 g/mm BLZ=500 nm grating. Raman spectra were obtained after 30 s, 1 min, 5 min, 10 min and 30 min while keeping the applied potential at 1.6 V vs RHE.

The electrochemical cell consisted of catalyst deposited on a GCE (Pine Research), Pt electrode, and Hg/HgO electrode (Pine Research) as WE, CE, and RE electrodes, respectively. N_2_ saturated 1.0 M KOH electrolyte was used in all the experiments. The laser beam was focused on the working electrode during the in‐situ measurements.

## Results and Discussion

### Structural and Physicochemical Characterization

Figure S1 shows the PXRD pattern and Rietveld refinement of the base material (BGLC587), which has the same structure and phase composition of the commercially available analogue used in our previous study.^[11]^ Briefly, we mainly see the rhombohedral LaCoO_3_ phase and two minor orthorhombic phases of the double perovskite BaGdCo_2_O_6‐δ_ and the single perovskite phase Gd_0.8_La_0.2_CoO_3_ (GdFeO‐type structure). Subsequently, we moved forwards with substituting Co by Fe and Ni and the synthesized compositions are summarized in Table S1.

**Figure 1:**
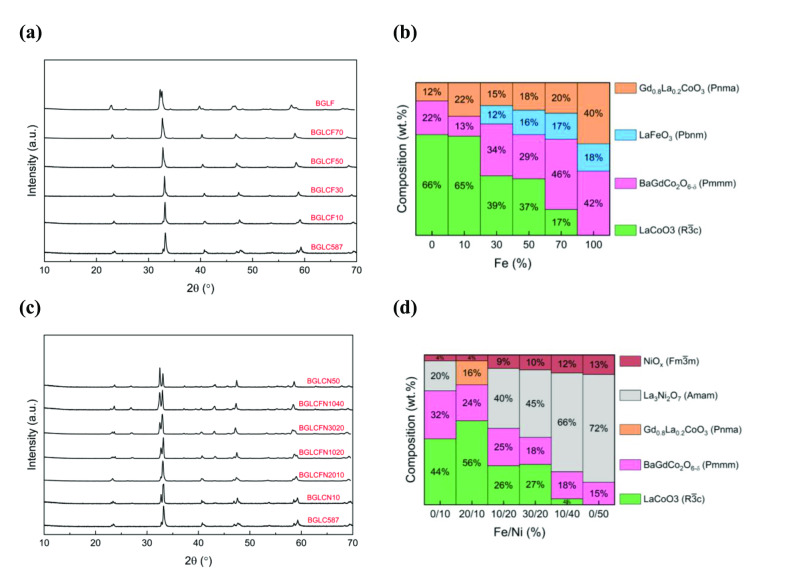
PXRD data of the twelve synthesized BGLC587‐based compositions, substituted by Fe and Ni. **(a)** PXRD patterns of as synthesized Fe‐substituted BGLC587. **(b)** Phase composition of BGLC587 as a function of Fe substitution. **(c)** PXRD patterns of as synthesized Fe‐ and Ni‐substituted BGLC587. **(d)** Phase composition of electrocatalysts with both Fe and Ni substitution. The x‐axis gives the percentage of Fe and Ni on B‐site in BGLC587. Ni content increases from left to right.

The PXRD patterns of the Fe substituted BGLC587 are given in Figure 1a, where we observe a slight shift towards lower 2θ values of all peaks with increasing Fe content. As expected, this is accompanied by an increase in the unit cell volume of all the main phases with increasing Fe‐content (Figure S2a). This phenomenon has been observed in other Co‐containing perovskites,[^19^, ^26^] where the increase is due to the larger covalent radius of Fe^3+^ (both low‐ and high‐spin configurations) compared to that of Co^3+^. There is also a small peak occurring at approximately 26° 2θ, which is due to an increase in the orthorhombic (single) perovskite structure with Fe.

In Figure 1b and after Rietveld refinement we observe that there are four different phases involved in the phase compositions when the Fe content is larger than 30 %. These are: rhombohedral LaCoO_3_ (LC) and the three orthorhombic phases BaGdCo_2_O_6‐δ_ (BGC), LaFeO_3_ (LF) and Gd_0.8_La_0.2_CoO_3_ (GLC). We note that the latter two phases have quite similar symmetry (Pbnm/Pnma) and that a good fit from Rietveld refinement was usually obtained without the LF phase. Therefore, we do not exclude the fact that some of the GLC phase can be mistaken for the LF phase. It is also evident that the amount of the LC phase decreases with increasing Fe content, while the BGC, GLC and LF phases increase. From this, it seems that increasing Fe‐content favors the formation of the double perovskite phase (BGC) and that the rhombohedral symmetry of LC is transformed to orthorhombic as the Co‐content declines. This agrees with other relevant findings in literature.[^26^, ^27^]

In the case of the compositions containing Ni and a combination of Ni and Fe, we can see the emergence of new diffraction peaks in the PXRD (Figure 1c), therefore the formation of new phases. Rietveld refinement of these samples revealed the formation of two new phases, the orthorhombic Ruddlesden‐Popper (RP) type La_3_Ni_2_O_7_ (n=2) and the cubic NiO_x_ phase. The orthorhombic GLC phase disappears when 10 % Ni is introduced and the LC phase is reduced, though maintaining its unit cell volume (Figure S2b). As more Ni is introduced, the LC phase decreases and finally disappears, the NiO_x_ increases, while the RP‐phase dominates with 72 wt.%. It is highly likely that the formation of NiO_x_ is a consequence of excess Ni, because the RP‐phase has a 3 : 2 ratio between A‐ and B‐site cations, and the desired BGLC‐phase has a 1 : 1 ratio. No secondary phases with Co were found, indicative of Ni being the only excess B‐site cation.

In general, Ni seems to have little effect on the unit cell volume of the BGC phase, therefore the observed changes must be related to the increasing Fe content. On the other hand, Ni affects the unit cell of the LC phase, which increases with increasing Ni‐content, as was also seen with Fe substitution. Huang, Lee and Goodenough also reported structural parameters indicating an increase in unit cell volume of LaCoO_3_ with 40 at.% Ni.^[28]^

The macro‐ and micro‐structural properties of the BGLC587‐based materials, such as porosity and grain size have been assessed with secondary electron SEM imaging, while the elemental quantification has been studied by EDS in both SEM and STEM. The results are presented in Figures S3–S5 and Table S2 in the SI. The base material, BGLC587, was determined to have a macroporous structure with pore diameters above 50 nm. A large grain size distribution is also observed with their sizes ranging from 10 nm and up to 500 nm. It is also clearly visible that the smaller nanoparticle grains are localized on the surface of the larger grains. Some intermediate sized grains are randomly distributed. As Fe is introduced in the structure it is seen that the porosity increases by decreasing the pore diameter and increasing their population. Iron impacts the grain size too and the overall trend is that the grain sizes decrease with the larger ones becoming less faceted.

In the case where Ni and Ni/Fe are introduced, the change in the porosity is not as evident as with only Fe. Almost all the macrostructures look similar with the exception of BGLCN50 and BGLCFN2010, which might have a higher surface area than the others. As for the grain sizes, it seems that Ni in general increases the grain size when compared to the base material.

The SEM‐EDS results (Table S2) show no elemental impurities in any of the synthesized samples, while the quantification of each element matches very well the nominal molar fractions in each composition. Elemental analyses with STEM‐EDS on selected compositions, show that all the elements are present in substantial amounts and homogeneously distributed in the investigated grains. Therefore, we can safely conclude the successful synthesis of all the compositions and below we continue with the electrochemical characterization and performance of the material for the OER in 1.0 M KOH.

### Electrochemical Characterization

The electrochemical analysis starts with LSV as well as Tafel analysis as means of general activity indicators. These will be further analyzed in terms of intrinsic catalytic activity, by decoupling surface area effects.

**Figure 2:**
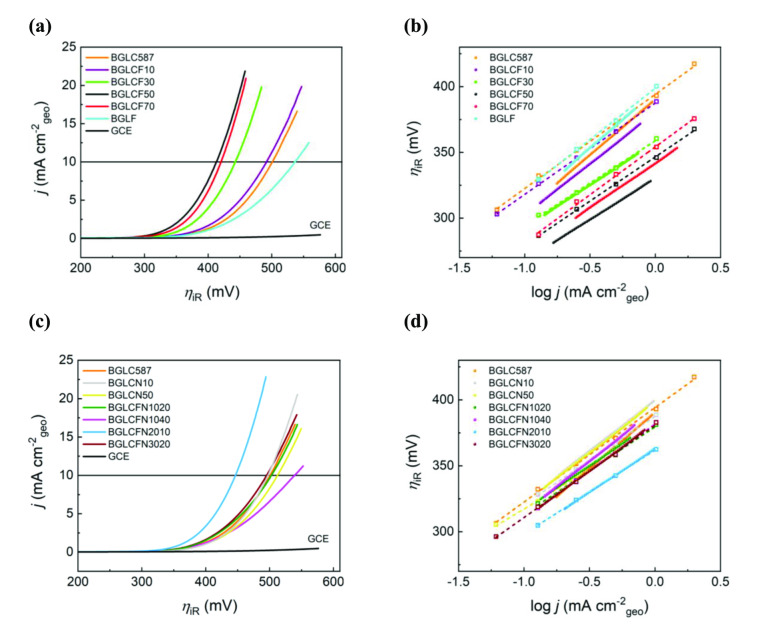
Electrochemical activity of the Fe and Ni substituted BGLC587 in O_2_ saturated 1.0 M KOH using the calibrated SCE reference electrode. **(a)** iR corrected LSVs collected at 10 mV s^−1^ and **(b)** corresponding Tafel slopes of Fe substituted BGLC587. **(c)** and **(d)** similar for the Ni‐ and Fe‐substituted BGLC587. Dotted lines and open square symbols correspond to Tafel slopes from CP (also iR corrected), while the solid lines correspond to Tafel slopes from LSV. The iR correction procedure can be found in Supplementary Note 2 in the SI.

Figure 2a and c present the LSV curves for the Fe‐ and Ni‐substituted BGLC587 electrocatalysts. Regardless of any surface area effects at this point, it is evident that the Fe‐substituted BGLC587 greatly improve the performance of the base material, while Ni substitution has little effect, except for some increase seen in BLCFN2010 (20 % Fe and 10 % Ni). Complete Co substitution by Fe is also detrimental (see BGLF), indicating the importance of a certain Co content. This can also be correlated with the low intrinsic OER activity of Fe oxides estimated by Trasatti′s volcano curves and M‐OH bond strengths.^[29]^

**Figure 3:**
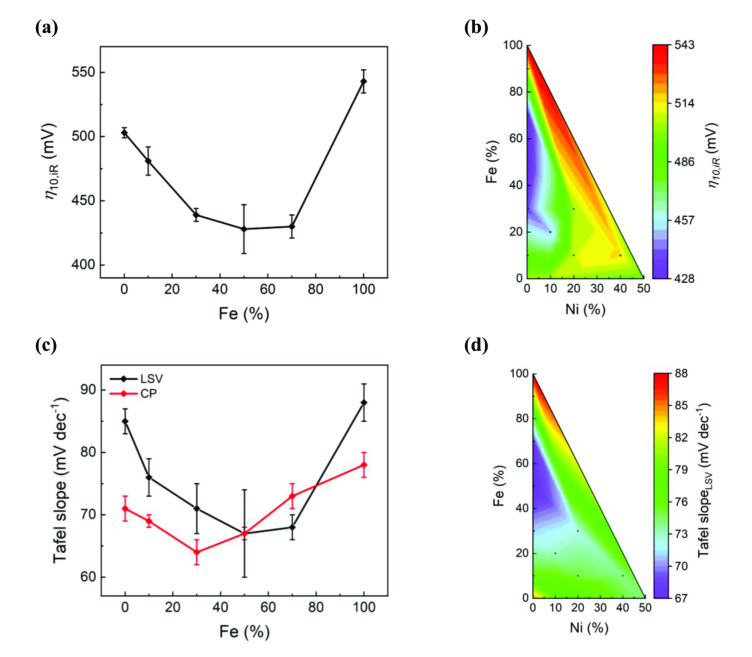
Overpotentials and Tafel slopes of all samples in contour plots. **(a)** Overpotentials at 10 mA cm^−2^_geo_ for the Fe‐substituted BGLC587. **(b)** Contour plot of the overpotentials at 10 mA cm^−2^_geo_ for all the B‐site substituted BGLC587. **(c)** Tafel slopes of the Fe‐substituted BGLC587 from both LSV and CP measurements. **(d)** Contour plot of the Tafel slopes of all the B‐site substituted BGLC587. All values are mean values from three independent measurements, except for Tafel slopes from CP, which are from a single measurement.

A better overview of the overpotentials at 10 mA cm^−2^_geo_ is given in Figure 3, where we observe that the overpotentials reach a minimum value of 428 mV at 50 % and 70 % Fe content. The highest activity lies close to the Fe‐axis (Fe‐substituted BGLC587 s), except for BGLCFN2010, which has an overpotential of 451 mV that is close to the best performing BGLCF50 and BGLCF70. BGLCFN2010 is also the only material with Ni that contains no RP or NiO_x_ phases, which means Ni is incorporated successfully into the perovskite structure. This is an indication that low Ni content in combination with Fe increases the OER activity, whereas higher content has the reverse effect. In comparison, She et al. found that 10 % Ni substitution on B‐site in Sr_0.95_Ce_0.05_FeO_3‐δ_ (SCF) resulted in an increased OER performance, arguing that Ni increases the electronic interaction between active sites, facilitating the OER.^[30]^

From the Tafel curves in Figure 2b and d, we observe that the Tafel region spans below one order of magnitude of current density when derived from LSVs, but above one order of magnitude when derived from CP. This is expected, since in CP mode steady‐state conditions are achieved, while LSVs taken at 10 mV s^−1^ are not true steady‐state conditions as there will be a contribution from capacitive currents^[31]^ (see Supplementary Note 3 in SI for CP curves and discussion). Nevertheless, a comparison between LSV‐ and CP‐derived Tafel slopes show a similar trend (Figure 3c), indicating that Fe substitution in the range between 30 % and 70 % (Figure 3d) improves the catalytic activity of BGLC587, while fully Co replacement by Fe is indeed detrimental to the activity.

The improved OER activity of these Fe substituted perovskite cobaltites fits well with the previously reported “Fe effect”.^[4]^ Although, several of the articles explaining why Fe increases activity are contradictory, some of them are in agreement with the findings of this work. One key effect of introducing Fe in BGLC is that the Fe−O bond strength is larger than that of Co−O. As found by Lim et al., introducing Fe in the similar perovskite PrBa_0.8_Ca_0.2_Co_2_O_6‐δ_ evidently resulted in less oxygen vacancies in the lattice.^[19]^ Considering that BGLC is already acceptor doped the increased O‐content will lead to an increased electron hole concentration localized at either Co or Fe as they become tetravalent cations. Such an increased electron hole concentration should aid the oxidation of water at the anode.

Ex‐situ XPS measurements of the Fe‐substituted BGLC587 s in this work found no shift in the Fe 2*p* binding energy (Figure S6a) and hence no tetravalent Fe could be identified. On the other hand, the intensity of the O 1s binding energy peak (Figure S6b) increased with Fe content and supports the abovementioned theory that Fe binds O more strongly. For comparison, work by Li et al. found that the Tafel slope of an CoFeO_x_ alloy followed the Fe^4+^ content during OER and a peak activity was reported in the 40–60 % Fe alloying range, similar to the BGLCF series in this work. Li et. al suggested that Fe^4+^ acts as a redox cooperative center increasing the oxidizing power of a Co : Fe site compared to a single Co^4+^ site.^[32]^ It is likely that Fe^3+^ in BGLCF is oxidized to Fe^4+^ at OER potentials and thus the similarity to the work by Li et al. becomes apparent. Nevertheless, this would have to be confirmed by further measurements such as e. g. operando ambient pressure XPS.

### Intrinsic Activity

Next, we expanded our electrochemical analyses to evaluate whether the increased electrochemical activity of the Fe‐ and Ni‐substituted BGLC587 is intrinsic or not. For this, we decoupled any surface area effects and we determined the latter with three primary methods, namely BET surface area, double layer capacitance (*C*_dl_) extracted from CV (at non‐faradaic potentials) and EIS (at three potentials including non‐faradaic, onset and 10 mA cm^−2^) and finally by calculating the *R*_ct_ *C*_dl_ product.^[23]^ As there is no consensus of which one is the most appropriate, we present a comprehensive comparison of those and we conclude that the best one is through the *R*_ct_ *C*_dl_ product in the OER potential regime. The *R*_ct_ *C*_dl_ product (with unit in seconds (s)) is an indicator of the ICA of a catalyst, as the total charge transfer resistance (*R*_ct_) calculated by EIS of a studied reaction is normalized by the double layer capacitance (*C*_dl_). The latter is analogous to the ECSA of the catalyst, which can also be found by EIS at any potential regime. We further use the inverse form, (*R*_ct_ *C*_dl_)^−1^, with unit s^−1^, which then reflects the turnover frequency (TOF),^[33]^ of the catalyst and the higher its value the higher the ICA of the catalyst.

**Figure 4:**
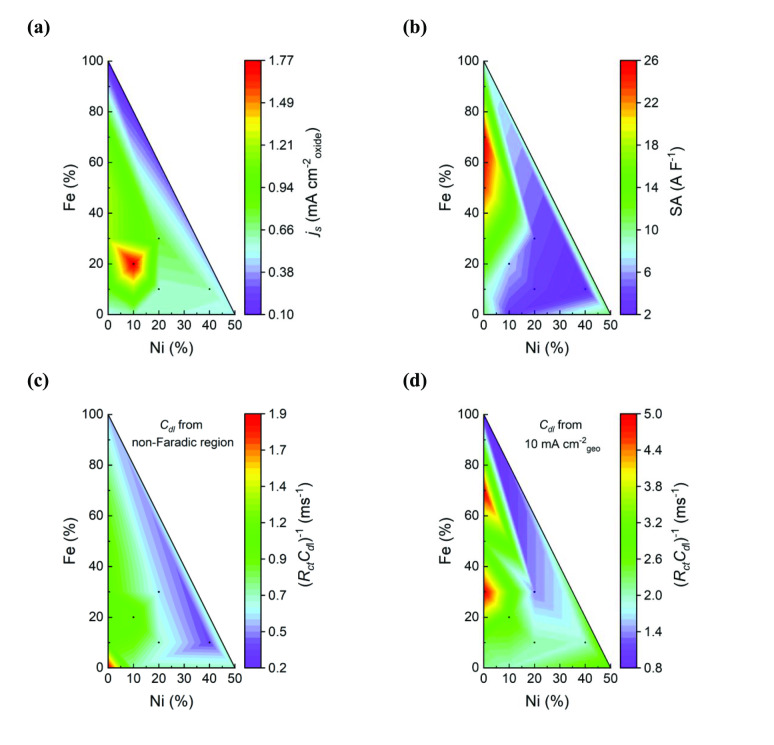
Contour plots of intrinsic activities of all B‐site substituted BGLC587. **(a)** Specific activity at 1.63 V vs RHE of all B‐site substituted ECs. Current is normalized by the BET surface area and **(b)** by the double layer capacitance from EIS in the non‐faradaic potential region. **(c)** and **(d)** Contour plots of the (*R*_ct_ *C*_dl_)^−1^ products (*R*_ct_ at 10 mA cm^−2^_geo_) of all B‐site substituted catalysts, with *C*_dl_ from EIS in the non‐faradaic potential region and at 10 mA cm^−2^_geo_ respectively.

The results from the surface area measurements used for assessing the ICA of the Fe‐substituted BGLC587 samples are summarized in Figure S7 and Table S3. Similar measurements were performed for the Ni‐substituted BGLC587 samples and the contour plots of Figure 4 include all the twelve compositions.

BET measurements show that the surface area in the Fe‐substituted BGLC587 increases with increasing Fe content almost linearly. On the other hand, BGLF is amongst the most underperforming catalysts, and this is seen in its low specific activity in Figure 4. EIS measurements and *C*_dl_ determination in the non‐faradaic region corresponds well to the *C*_dl_ found by CV, while specific activities found through BET and *C*_dl_ from EIS in the non‐Faradaic region are quite different with almost opposing results as seen in Figure 4a and b. The contour plots in Figure 4c and d, show that the two (*R*_ct_ *C*_dl_)^−1^ products also vary with Fe and Ni contents. Figure 4c predicts the highest ICA for BGLC587 and BGLCFN2010 and that high Ni content is detrimental to the activity, which is not the case for BGLCN50 for example. Figure 4d on the other hand, predicts increasing ICA with increasing Ni content up to BGLCN50 and Fe content up to 70 %. It also predicts that BGLF (100 % Fe) is detrimental to the activity, which is true, a fact that wasn′t predicted through the (*R*_ct_ *C*_dl_)^−1^ product in the non‐faradaic region (Figure 4c). Overall, we find that the (*R*_ct_ *C*_dl_)^−1^ product in the OER regime correlates well with the previously observed electrochemical activity and represents a suitable “operando” activity descriptor at potentials in the OER regime. This finding further exemplifies the importance of EIS and its applicability in decoupling surface area even at potentials where faradaic processes occur.

### Mechanistic Insights

**Figure 5:**
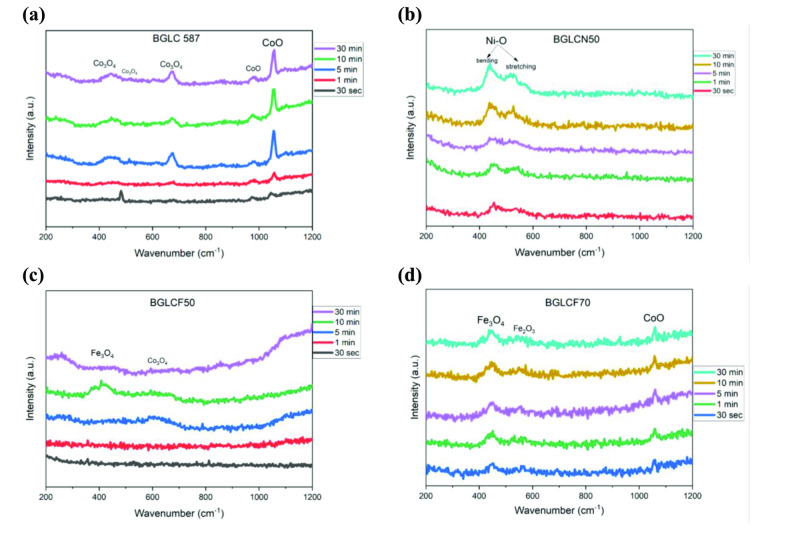
In‐situ Raman spectra collected after 30 s, 1, 5, 10 and 30 min of OER operation at 1.6 V vs. RHE in 1.0 M KOH for **(a)** BGLC587 **(b)** BGLCN50, **(c)** BGLCF50 and **(d)** BGLCF70.

**Figure 6:**
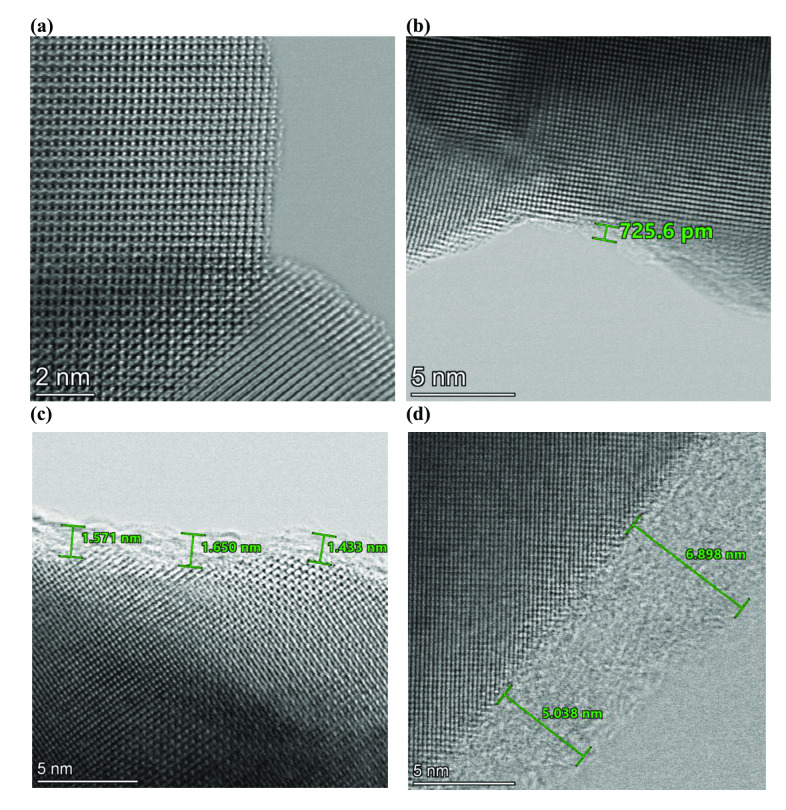
STEM investigations of BGLCF70 showing BF STEM images at different time points under OER at 10 mA cm^−2^ after **(a)** 0 min, **(b)** 30 s, **(c)** 2 min, **(d)** 10 min.

Figure 5 shows the obtained in‐situ Raman spectra from BGLC587, BGLCN50, BGLCF50 and BGLCF70 during OER at 1.6 V vs. RHE. We see that the different peak intensities vary initially with time and stabilize typically after 10 min. This indicates the restructuring of the catalysts especially during the first 10 min under OER conditions. We have previously observed the surface amorphization in BGLC587,^[11]^ a very common phenomenon in perovskite‐based catalysts.^[34]^ Surface amorphization is also observed in this work as it can be seen in the BF STEM images of BGLCF70, acquired at certain points (0, 30 s, 2 and 10 min) under OER conditions (Figure 6). The BF STEM images clearly show the development of the amorphous surface layer with time, matching to some extent the evolution and increase in intensity of the different Raman peaks, which are indicative of the occurring surface restructuring.

As proposed by May et al., the surface amorphization may be caused by an O *p*‐band center higher than −2.2 eV relative to the Fermi level, which can in turn lead to Fermi level pinning of the O *p*‐band and hence facilitate redox with both cations and lattice oxygen.^[34]^ Related to our materials, computational investigations by Mefford et al. have shown that the Ba_0.5_Gd_0.5_CoO_3_ has an O *p*‐band center higher than −2.2 eV. Based on this, we highlight that the observed amorphization could be a result of the OER proceeding through the lattice oxygen mechanism (LOM).^[36]^ This mechanism is observed in perovskites with acceptor dopants on the A‐site, where the energy of the metal 3*d* band is lowered and the metal 3*d*/O 2*p* covalency is increased. Based on previous observations of surface amorphization in BGLC587 and since all catalyst in this work (except the Ni‐substituted ones) will have the same A‐site composition, we can assume that surface amorphization likely happens in all of them.

The Raman shifts for BGLC587, BGLCF50 and BGLCF70 correspond to oxides of Co and Fe, but not to (oxy−)hydroxide. This suggests that the OER mechanism in these catalysts possibly proceeds through the reaction of two adjacent oxy intermediates.^[1]^ As the Tafel slopes in these catalysts approach 60 mV dec^*−*1^ (2RT/F) (Figure 3), the rate determining step (RDS) could be the deprotonation of the adsorbed hydroxyl and formation of water in the Krasil′shchikov path as shown in Equation 2.^[1]^

(2) $$MOH+{OH}^{-}\to MO^{-}+H_{2}O$$

As not all Tafel slopes are close to 60 mV dec^−1^ and in addition to the fact that different phases (Figure 1) may have different RDSs, it is possible that the observed Tafel slopes are the average product of several ones. Interestingly, the lowest Tafel slopes for the Fe‐substituted materials correspond to those with the highest amount of the double perovskite phase (excluding BGLF which is clearly inferior), which may support this theory further.

Moreover, the observed Raman shifts indicate also that both di‐ and tri‐valent Co and Fe are present at the surface of the electrocatalysts, however no indications of tetra‐valent Co or Fe were found. This further suggests the participation of the lattice oxygen (LOM) in the OER mechanism, in which the transition metal is not changing its oxidation state.[^36^, ^38^] Although, we keep in mind that this result alone cannot unambiguously exclude the AEM.

On the contrary, in‐situ Raman measurements with BGLCN50, show the formation of (oxy−)hydroxides (NiOOH) indicating the presence of Ni(III), which can be correlated with the mixed valence of Ni(II/III) in the dominating La_3_Ni_2_O_7_ phase observed in BGLCN50 in Figure 1. Although, the response can also be attributed to the secondary NiO_x_ phase, which typically transforms into oxy‐hydroxide during OER in alkaline solution.^[39]^ Zhang et al. calculated the O *p*‐band center for a series of B‐site substituted La_3_Ni_2_O_7_ and found that it was mainly lying below −2.2 eV,^[40]^ therefore surface amorphization might be suppressed in BGLCN50. Based on this and the Raman results, we can conclude that the OER mechanism is different in BGLCN50 and most likely proceeds through the formation of terminal peroxide groups, possibly following the AEM mechanism and not the LOM. As the Tafel slope of BGLCN50 is close to 60 mV^−1^ dec, it could be suggested that the reaction proceeds through the 4th step in the Shinagawa path (Equation 3).^[1]^

(3) $$MOOH+OH^{-}\to MO_{2}^{-}+H_{2}O$$

### Stability and Faradaic Efficiency

The stability of some of the catalysts (we selected both efficient and non‐efficient ones according to the ICA results from the (*R*_ct_ *C*_dl_)^−1^ product) was studied galvanostatically at 10 mA cm^−2^. It is noted that for these measurements, a Hg/HgO (20 % KOH) reference electrode was used instead of the SCE. The results are summarized in Table 1 and Figures S8–S10 and they collectively show that the stability follows the activity in the Fe‐substituted BGLC587. This can also be seen in Figure S9, where there is the same trend between overpotential and Tafel slopes and stability with Fe content. It was again interesting to see that 100 % Co substitution with Fe, as well as high Ni substitution without Fe showed inferior stability in addition to the low activities determined from ICA.

**Table 1:** Data on the stability of the selected catalysts as obtained from Figure S7.

Electrocatalyst	10 % degradation (h)	Overpotential at 10 % degradation (mV)	20 % degradation (h)	Overpotential at 20 % degradation (mV)
BGLC587	5.7	688	24.1	750
BGLCF50	11.2	632	∼ 50^*^	∼ 690^*^
BGLCF70	8	559	54.7	610
BGLF	3.4	763	∼ 26^*^	∼ 832^*^
BGLCN50	4	641	6.3	700

*CP measurement was cancelled close to the point of 20 % degradation. Therefore, a linear fit of the last 60 min of data points was used to extrapolate the curve.

We have also examined these catalysts post operation with XRD (Figure S10), directly on the C‐paper where the stability measurements were done. Interestingly, we observe that no new phases formed, and all catalysts maintained their initial phases after galvanostatic operation. This implies that the bulk structures of these materials are stable under operating conditions with no irreversible phase transitions occurring, therefore, it must be the amorphous surface layers that undergo further structural changes and dominate/dictate the catalytic activity of the materials. Finally, measurements of the FE at 5 mA cm^−2^ for the OER of selected catalysts show high efficiencies, with mean values ranging from 90 to 100 % (Figure S11). It is mentioned that the FE measurements were initiated after a dwell time of 20 min to reach steady state conditions. Moreover, as the N_2_ content was increasing during the measurements due to leakages, the reported FE values were corrected for it considering that air has a N_2_/O_2_ ratio of 78/21.

### Application in Unassisted, PV‐Driven Water Electrolysis

To test the applicability of our electrocatalysts in a full electrolysis cells, BGLCF50 was employed in a previously developed monolithic, PV‐driven electrochemical (PV‐EC) water splitting device. A thorough explanation of the PV‐EC design is given by Zhu et al.^[11]^ but in short, the monolithic device consisted of, (i) a NiMo cathode electrodeposited on Ti foil, (ii) a commercially available high‐performance triple‐junction GaAs photovoltaic cell (9.0x9.4 mm^2^ from Fullsuns Co.) and (iii) an anode with BGLCF50 deposited by pulsed laser deposition (PLD) on FTO‐coated glass, according to our previous work.^[11]^ We have previously found that the catalyst loading in such configuration was of the order of μg cm^−2^. We chose the BGLCF50 due to its high ICA as found through the (*R*_ct_ *C*_dl_)^−1^ product in Figure 4d, its high stability during the long‐term testing, accompanied by a high FE of almost unity.

**Figure 7:**
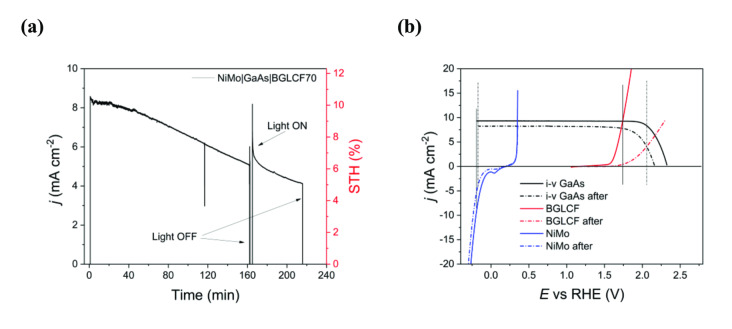
Application of BGLCF50 in a monolithic PV‐driven electrochemical water electrolysis cell. **(a)** Photocurrent density of the NiMo|GaAs|BGLCF50 PV‐EC cell under 1 sun solar simulated conditions in 1.0 M KOH. Light ON and OFF periods are also indicated. A blown‐up graph at around the 160 min mark is given in Figure S12. **(b)** LSVs of the NiMo cathode, GaAs PV and BGLCF50 anode taken before and after the chronoamperometric test in a).

The photocurrent density generated under 1 sun solar simulated conditions in 1.0 M KOH electrolyte is shown in Figure 7a. It is seen that the cell delivered an initial solar‐to‐hydrogen (STH) efficiency of approx. 10.5 %, which degraded to 9.5 % within 60 min of operation. The STH is further reduced to 5.1 % in just under 4 h of operation. Figure 7b shows the LSVs of the NiMo cathode, the GaAs PV and the BGLCF50 anode in the beginning and after 4 h of 1 sun solar simulated light. We observe that the degradation of the anode (red dashed line) contributes the most to the STH loss, in addition to a slight degradation of the GaAs PV. The latter is most probably related to moisture leakage through the sealing materials of the PV cell. The former is most probably related to the very thin catalyst layer (a few tens of nanometers) deposited on the FTO‐coated front glass. This is what we have observed previously,^[11]^ with the anode catalyst layer in the form of a thin film getting fully amorphous, a fact indicating the role and importance of an underlying crystalline phase. In a bulky form of the perovskite‐based catalysts, the retained crystalline phase offers most probably structural support and electronic conductivity to the top amorphous layer. To further investigate this point, additional studies and efforts are necessary by for example suppressing the extend of the amorphization and/or changing the cell configuration. In the latter, the catalyst can be placed adjacent to the PV in a bulky form. Although such a configuration will eventually increase the demand on land usage and mass loading of the catalysts, it may compensate by extending the lifetime of such monolithic devices.

Nonetheless, what is very important to notice is that the initial BGLCF50 catalyst has a perfectly matching overpotential with the maximum current density of the GaAs PV (solid black vertical line), making it an appropriate earth‐abundant catalyst material. In this configuration, there is no need for further improvement of the catalyst activity but rather of the catalyst stability. As already mentioned, this is particularly important in such thin film catalyst configurations (with catalyst loadings in the μg cm^−2^ range) to enable high performance, monolithic and stable PV‐EC devices with industrial scale perspectives.

## Conclusions

We have confirmed our main hypothesis that substitution of Co with Fe and Ni leads to an increased OER activity in our base material, BGLC587. The *R*_ct_ *C*_dl_ product serves as a suitable descriptor of the true intrinsic surface area‐normalized activity of electrocatalysts and indicated that a 30–70 % Fe content in BGLC587 led to the biggest increase in activity and stability. In‐situ Raman spectroscopy proved to be a powerful tool for mechanistic studies, and it provided valuable insights whether the OER mechanism follows the AEM or the LOM. Further mechanistic studies behind the amorphization mechanism and the role of the stable bulk phase are needed to design more stable and efficient catalysts in both powders, but also thin film configurations.

## Conflict of Interests

The authors declare no conflict of interest.

1

## Supporting information

As a service to our authors and readers, this journal provides supporting information supplied by the authors. Such materials are peer reviewed and may be re‐organized for online delivery, but are not copy‐edited or typeset. Technical support issues arising from supporting information (other than missing files) should be addressed to the authors.

Supporting Information

## Data Availability

The data that support the findings of this study are available from the corresponding author upon reasonable request.
