# Supplementary material for: Iron and Nickel Substituted Perovskite Cobaltites for Sustainable Oxygen Evolving Anodes in Alkaline Environment
Source: ChemSusChem. 2024 Nov 4;18(6):e202401403. doi: 10.1002/cssc.202401403 (PMC11912097; doi:10.1002/cssc.202401403)
Supplement: Supplementary file 1 — Supporting Information [file CSSC-18-e202401403-s001.pdf]

ChemSusChem

Supporting Information

Iron and Nickel Substituted Perovskite Cobaltites for Sustainable Oxygen Evolving Anodes in Alkaline Environment

Henrik Petlund, Alaa Faid, Junjie Zhu, Anuj Pokle, Truls Norby, Svein Sunde, and Athanasios Chatzitakis*

Supporting Information (SI)

Iron and Nickel Substituted Perovskite Cobaltites for Sustainable Oxygen Evolving Anodes in Alkaline Environment

Authors

Henrik Petlund,[1] Alaa Faid,[2] Junjie Zhu,[3] Anuj Pokle,[4] Truls Norby,[1] Svein Sunde,[2] Athanasios Chatzitakis[1*]

Affiliations

[1] Department of Chemistry, Centre for Materials Science and Nanotechnology, University of Oslo, Gaustadalléen 21, NO-0349 Oslo, Norway

[2] Department of Materials Science and Engineering, Norwegian University of Science and Technology (NTNU), N-7491, Trondheim, Norway

[3] Institute for Energy Technology (IFE), NO-2007, Kjeller, Norway

[4] Department of Physics, Centre for Materials Science and Nanotechnology, University of Oslo, POB 1048, NO-0316, Oslo, Norway

[*]Corresponding author: athanasios.chatzitakis@smn.uio.no

Keywords: alkaline water electrolysis, double perovskites, earth abundant catalysts, oxygen evolution reaction, in-situ Raman spectroscopy

List of Figures

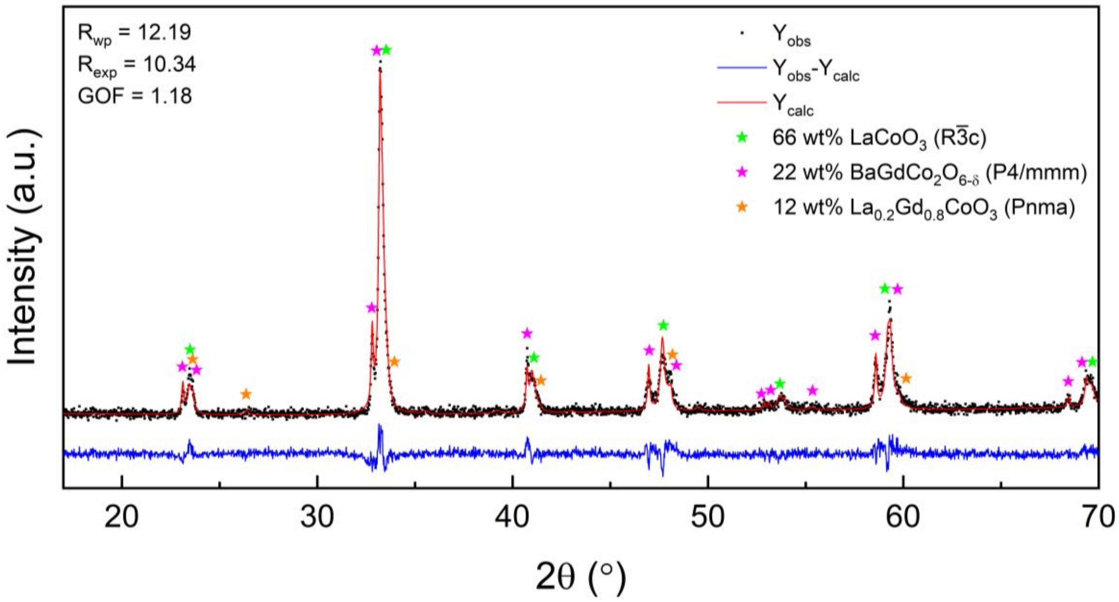

Figure S1: Powder XRD pattern of BGLC587 (black) with Rietveld refinement (red) and the residual plot (blue). Stars show peaks belonging to the refined phases.

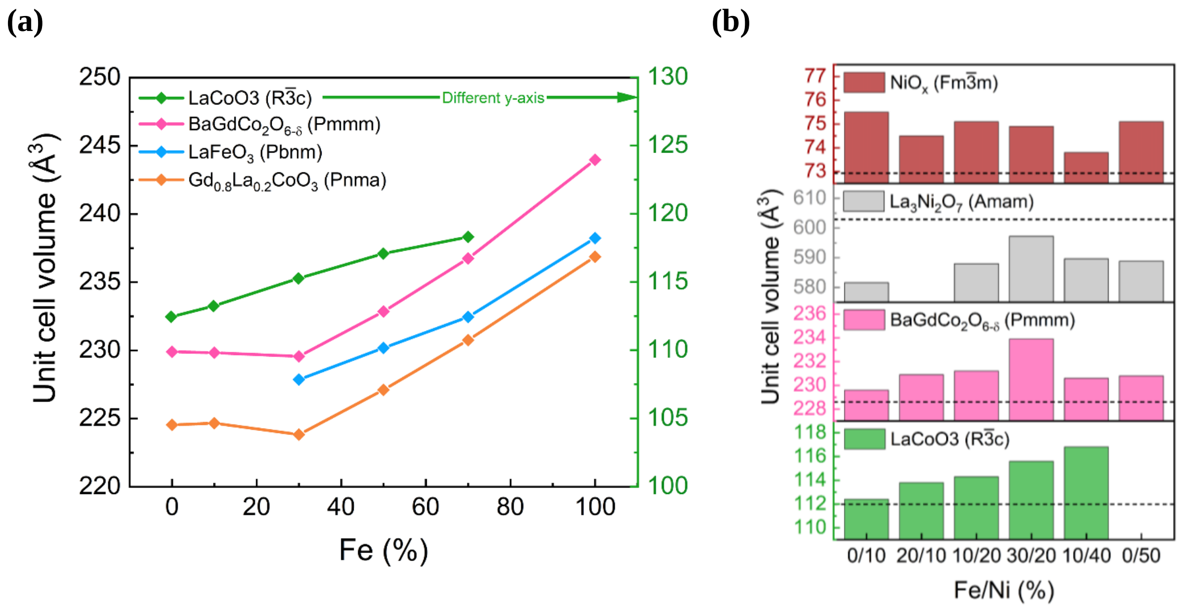

Figure S2: **(a)** Unit cell volume of the Fe-substituted phases. The green y-axis corresponds to the data points of $LaCoO_3$, whereas the other data follow the left axis. Both axes span 30 $Å^3$ to make comparisons between phases more obvious. The structural data were found by Rietveld refinement. **(b)** Unit cell volume of the Fe- and Ni-substituted BGLC587. The dotted lines represent the unit cell volumes reported in literature (found through the Inorganic Crystal Structure Database, ICSD). Experimental data were found by Rietveld refinement.

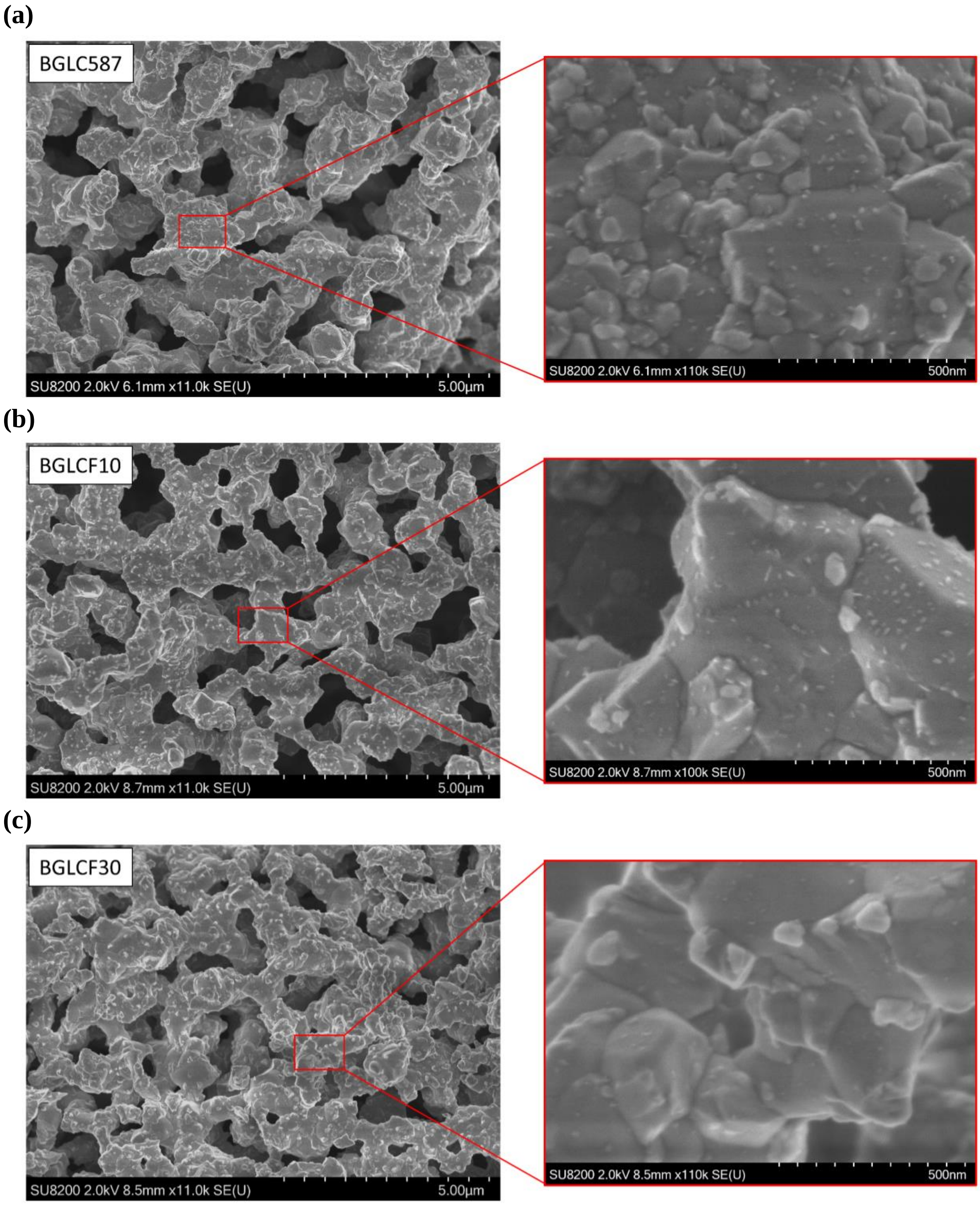
(a)
BGLC587
SU8200 2.0kV 6.1mm x11.0k SE(U) 5.00µm
SU8200 2.0kV 6.1mm x110k SE(U) 500nm
(b)
BGLCF10
SU8200 2.0kV 8.7mm x11.0k SE(U) 5.00µm
SU8200 2.0kV 8.7mm x100k SE(U) 500nm
(c)
BGLCF30
SU8200 2.0kV 8.5mm x11.0k SE(U) 5.00µm
SU8200 2.0kV 8.5mm x110k SE(U) 500nm

(d)

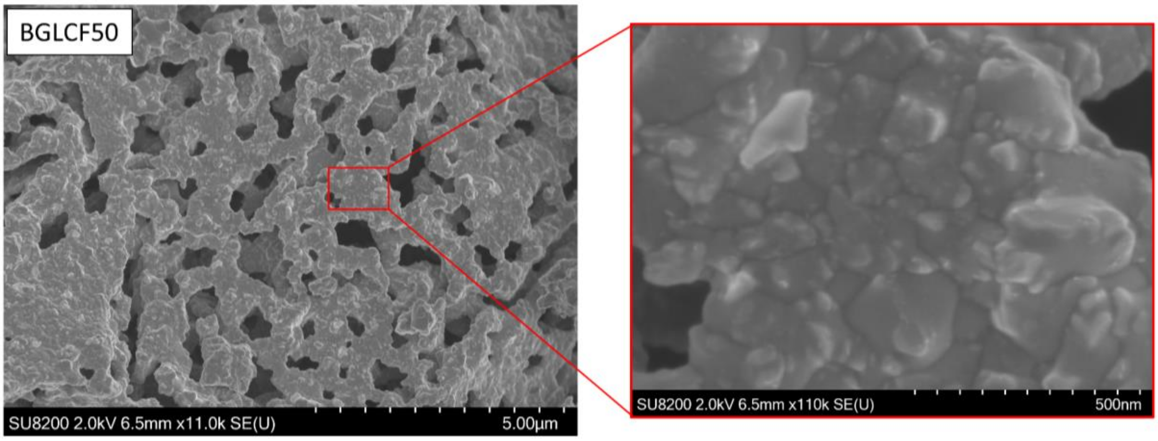

(e)

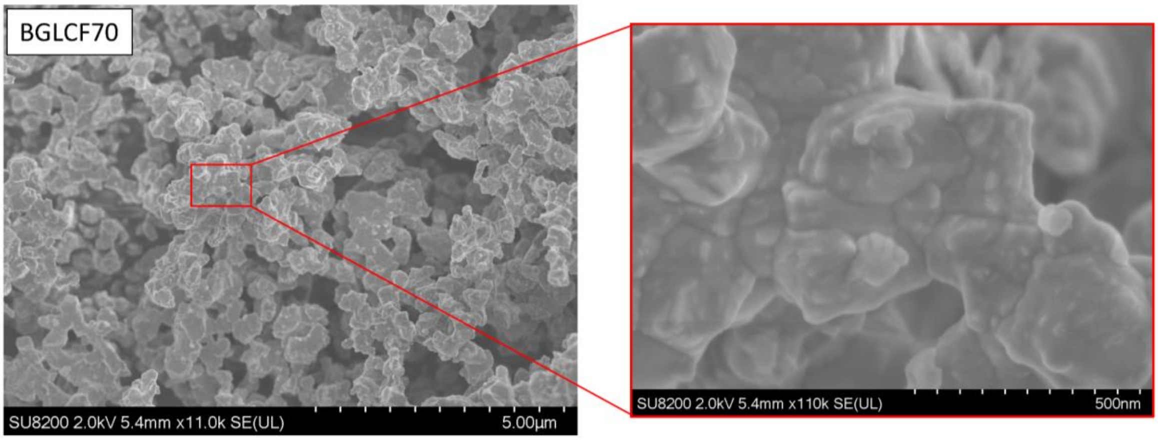

(f)

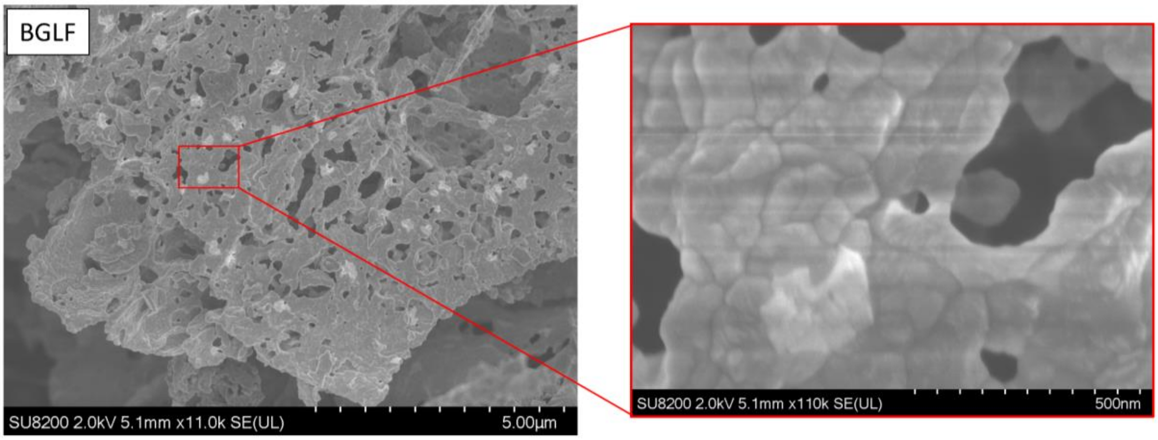

Figure S3: Secondary electron SEM micrographs of **(a)** BGLC587, **(b)** BGLCF10, **(c)** BGLCF30, **(d)** BGLCF50, **(e)** BGLCF70 and **(f)** BGLF.

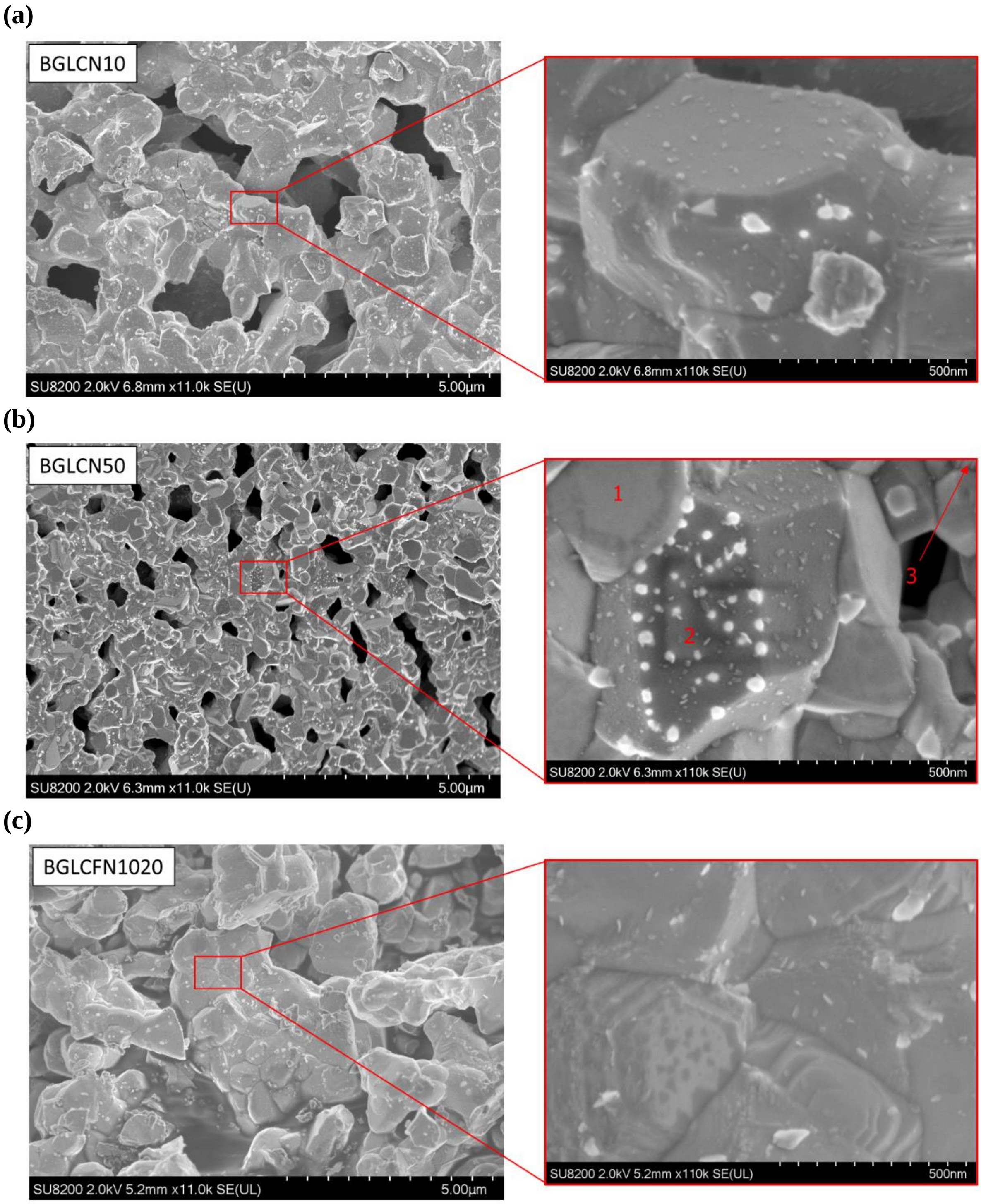
(a)
BGLCN10
SU8200 2.0kV 6.8mm x11.0k SE(U)
5.00μm
SU8200 2.0kV 6.8mm x110k SE(U)
500nm
(b)
BGLCN50
SU8200 2.0kV 6.3mm x11.0k SE(U)
5.00μm
1
2
3
SU8200 2.0kV 6.3mm x110k SE(U)
500nm
(c)
BGLCFN1020
SU8200 2.0kV 5.2mm x11.0k SE(UL)
5.00μm
SU8200 2.0kV 5.2mm x110k SE(UL)
500nm

(d)

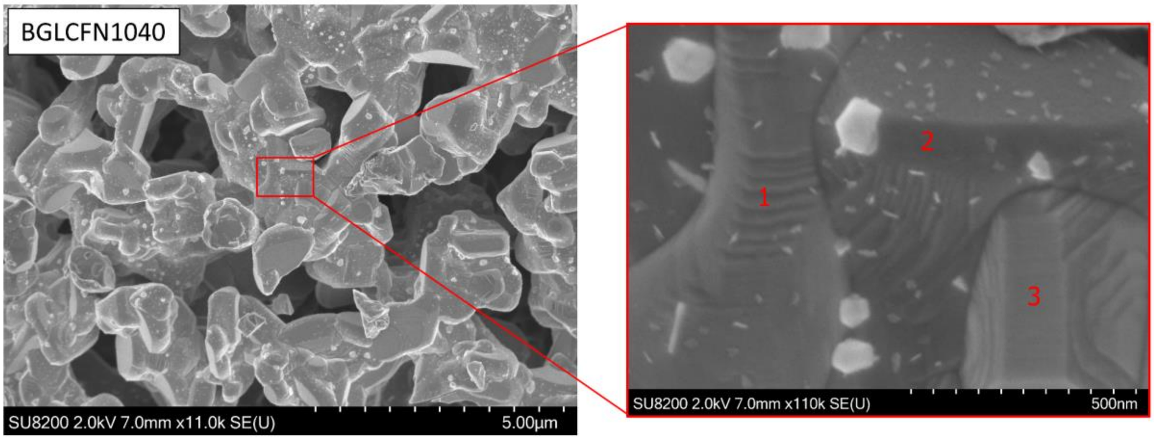

(e)

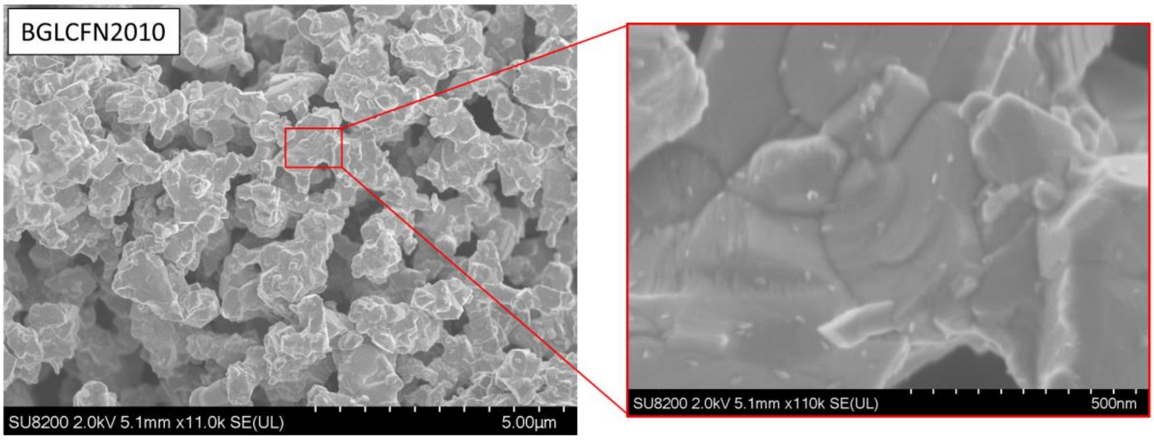

(f)

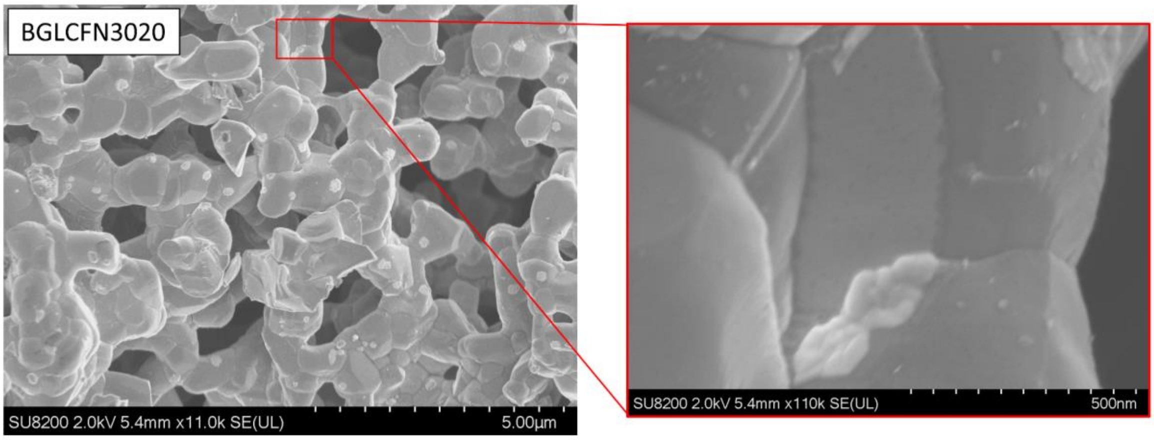

Figure S4: Secondary electron SEM micrographs of **(a)** BGLCN10, **(b)** BGLCN50, **(c)** BGLCFN1020, **(d)** BGLCFN1040, **(e)** BGLCFN2010 and **(f)** BGLCFN3020. Numbers 1, 2 and 3 indicate the location of Ruddlesden-Popper (RP), perovskite and NiO_x phases respectively observed with Z-contrast imaging.

(a)

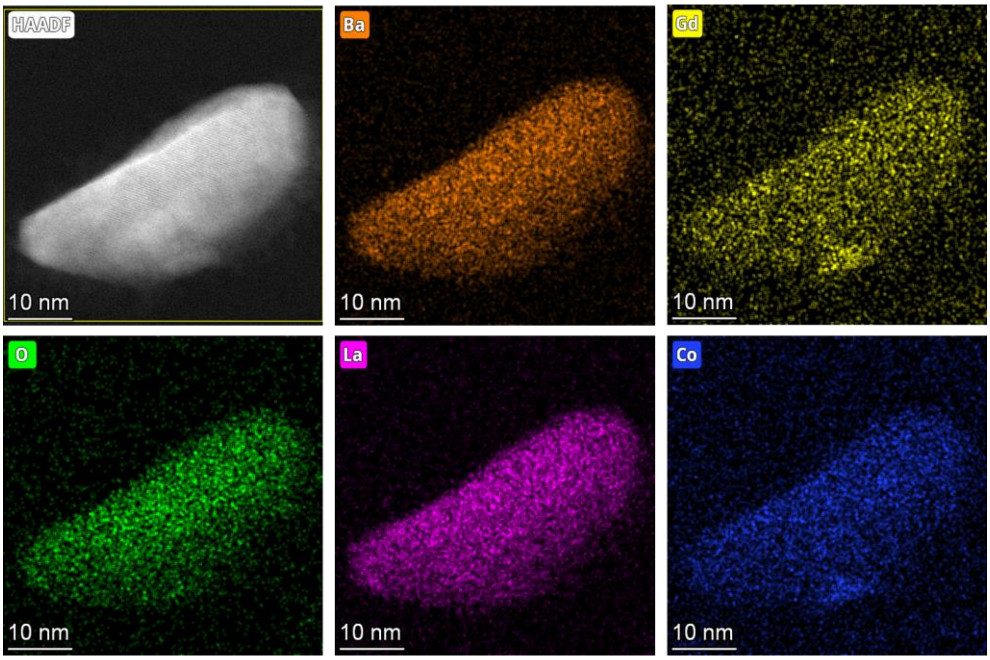

(b)

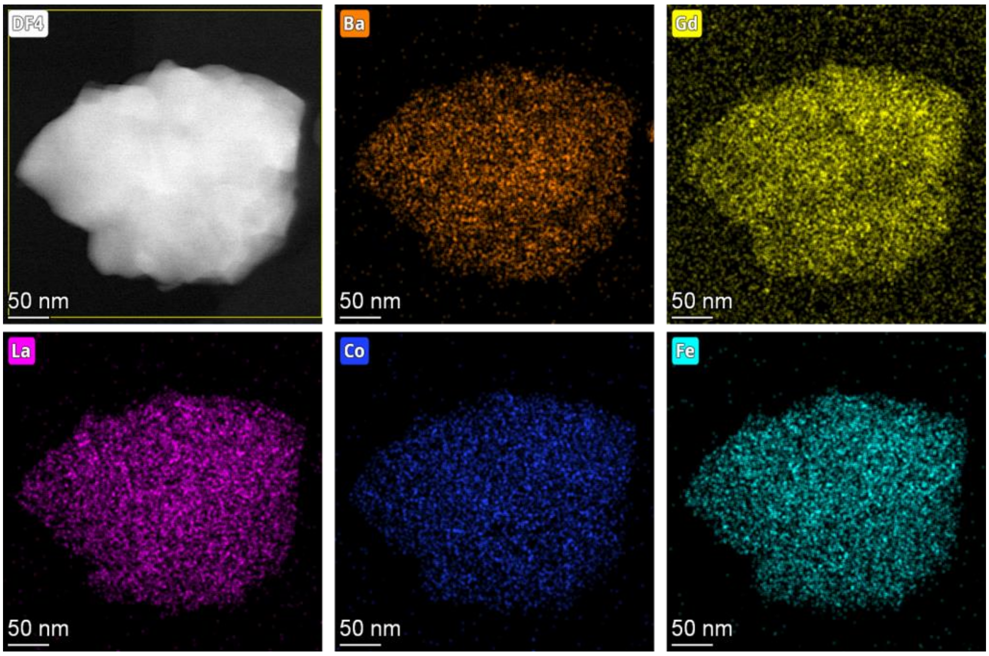

Figure S5: **(a)** STEM-EDS mapping of a particle in BGLC587. **(b)** STEM-EDS mapping of a particle in BGLCF70.

XPS spectra were taken on a Kratos Axis Ultra DLD spectroscope with monochromated Al Kα X-rays (hν = 1486.6 eV). High resolution spectra were obtained using pass energy (PE) 20 eV and step size 0.1 eV, while survey spectra at 160 eV and step size 1 eV.

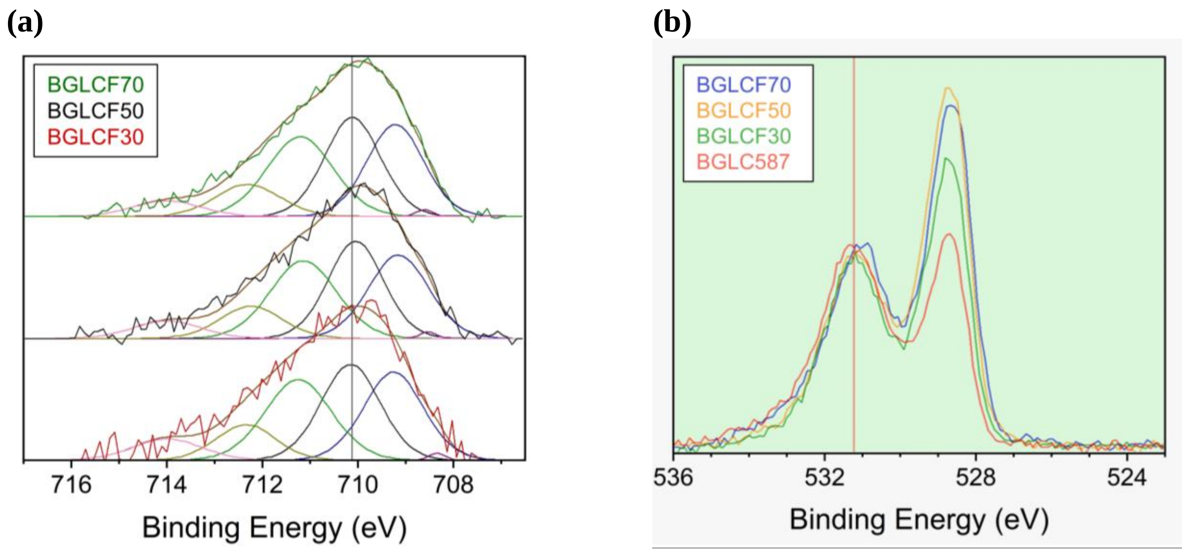

Figure S6: Binding energies found with XPS of **(a)** Fe 2*p* in BGLCF30, BGLCF50 and BGLCF70 and **(b)** O 1*s* in BGLC587, BGLCF30, BGLCF50 and BGLCF70.

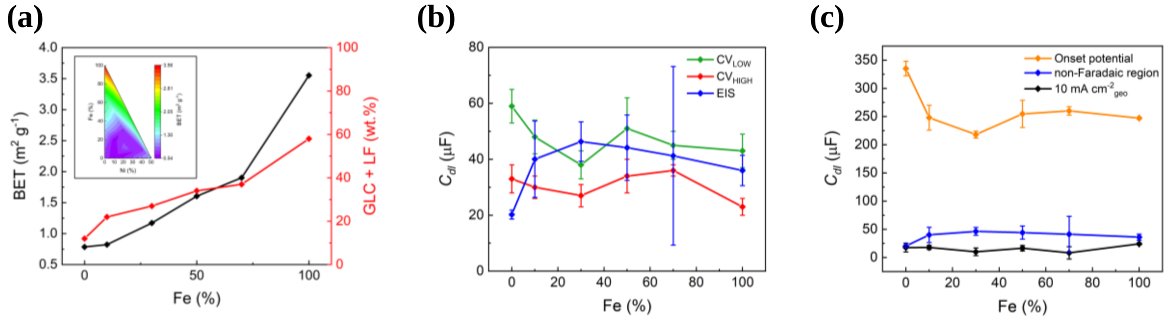

Figure S7: Surface area measurements of the Fe-substituted BGLC587 catalysts. **(a)** BET. **(b)** C_{dl} through CV measurements (at both low and high scan rates) in the non-faradaic region, including the one calculated through EIS at relevant potentials. **(c)** C_{dl} from EIS at the non-faradaic region, onset and at potentials for 10 mA $\mathrm{cm}^{-2}{}_{\mathrm{geo}}$.

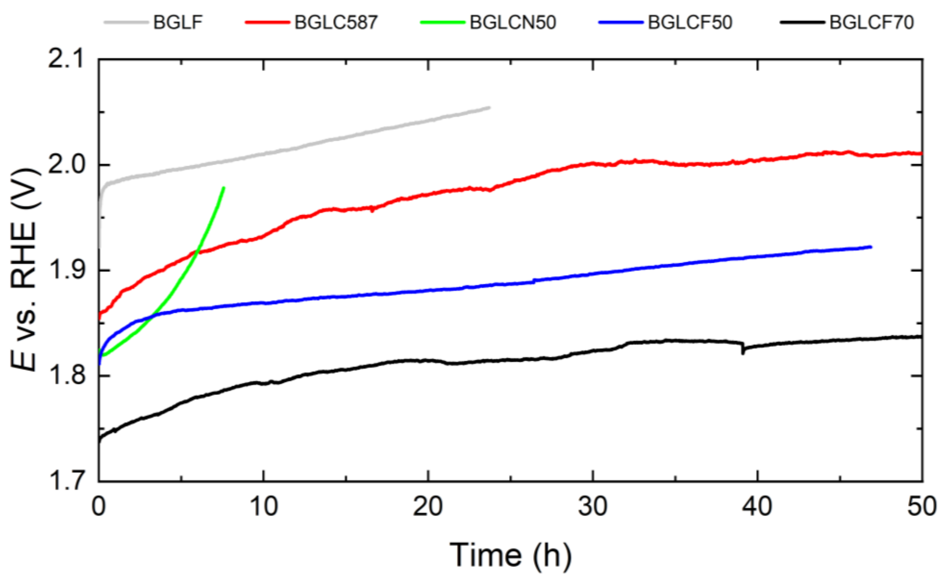

Figure S8: Chronopotentiometric (CP) curves of BGLF, BGLC587, BGLCN50, BGLCF50 and BGLCF70 electrocatalysts at 10 mA cm^{-2}_{geo} in O_2-saturated 1.0 M KOH. The working electrode was carbon paper with 5 mg cm^{-2} catalyst loading.

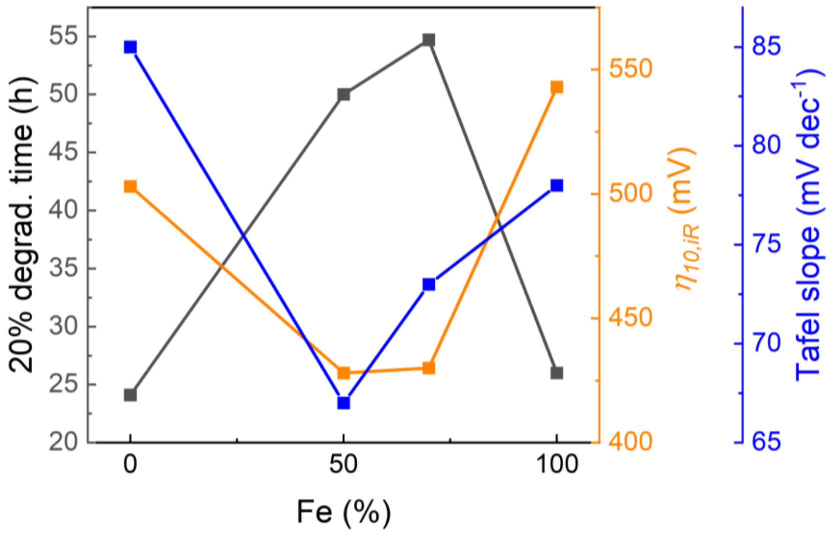

Figure S9: Stability vs activity trend. The stability (black line) is the time before reaching 20% degradation at 10 mA cm^{-2}_{geo}. The activity is given from the overpotentials at 10 mA cm^{-2}_{geo} and the Tafel slopes from CA.

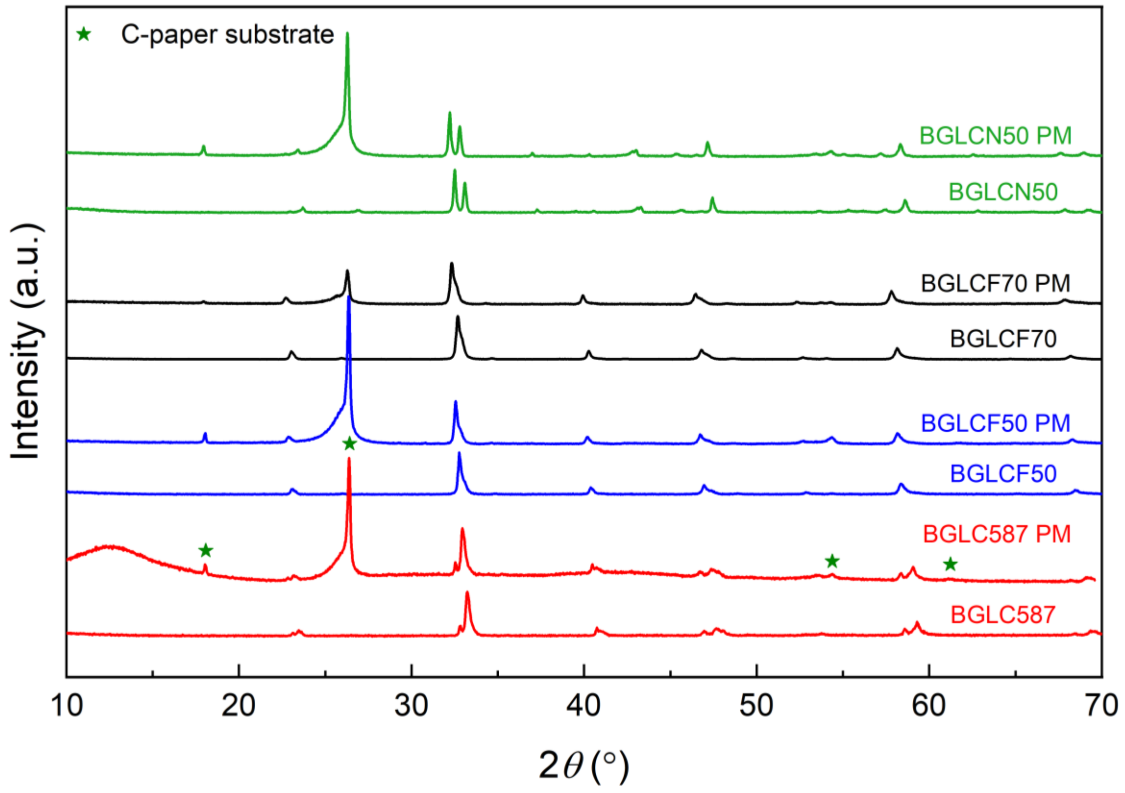

Figure S10: Post-mortem XRD patterns of BGLC587, BGLCF50, BGLCF70 and BGLCN50 with the respective as-synthesized powder XRD patterns.

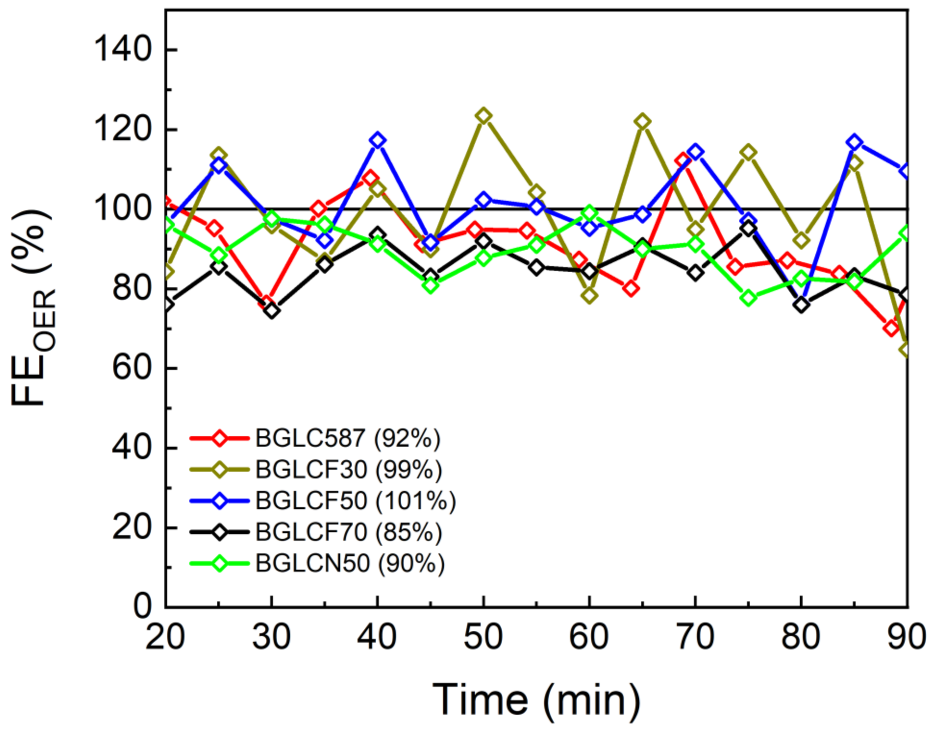

Figure S11: Faradaic efficiency at 5 mA cm^{-2}_{geo} for the OER of five selected electrocatalysts loaded on C-paper electrodes.

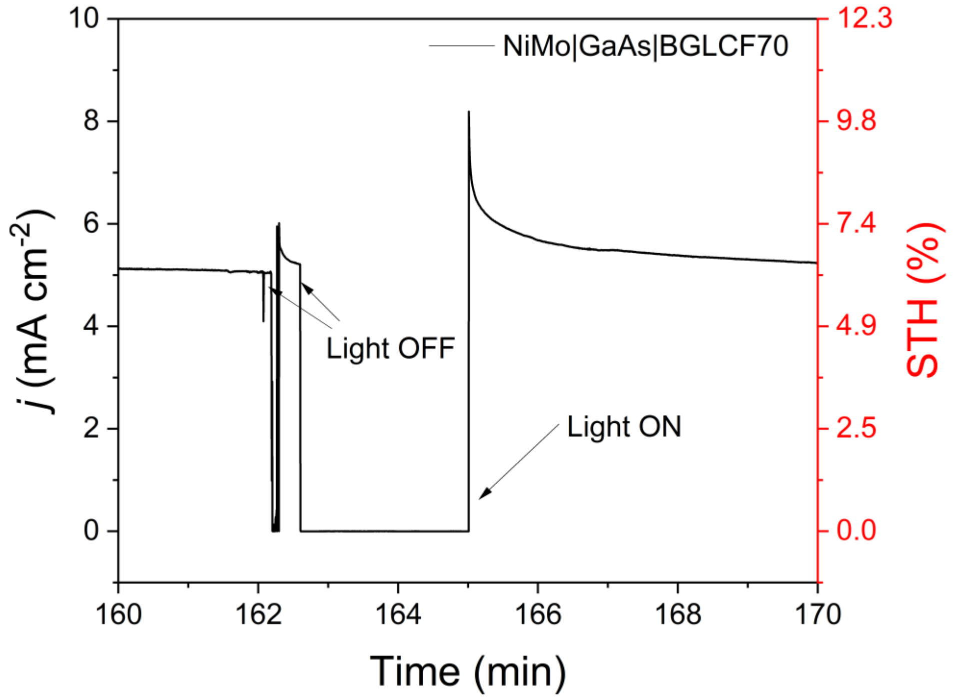

Figure S12: Blown-up graph of Figure 7a, indicative of the effect of light switching events at the indicated points.

List of tables

Table S1: Composition of the twelve synthesised electrocatalysts in this work with their respective abbreviations and nominal B-site substitution.

Stoichiometric composition	Abbreviation	Nominal substitution
$Ba_{0.5}Gd_{0.8}La_{0.7}Co_{2}O_{6-\delta}$	BGLC587	Base material
$Ba_{0.5}Gd_{0.8}La_{0.7}Co_{1.8}Fe_{0.2}O_{6-\delta}$	BGLCF10	B-site: 10% Fe
$Ba_{0.5}Gd_{0.8}La_{0.7}Co_{1.4}Fe_{0.6}O_{6-\delta}$	BGLCF30	B-site: 30% Fe
$Ba_{0.5}Gd_{0.8}La_{0.7}Co_{1.0}Fe_{1.0}O_{6-\delta}$	BGLCF50	B-site: 50% Fe
$Ba_{0.5}Gd_{0.8}La_{0.7}Co_{0.6}Fe_{1.4}O_{6-\delta}$	BGLCF70	B-site: 70% Fe
$Ba_{0.5}Gd_{0.8}La_{0.7}Fe_{2}O_{6-\delta}$	BGLF	B-site: 100% Fe
$Ba_{0.5}Gd_{0.8}La_{0.7}Co_{1.8}Ni_{0.2}O_{6-\delta}$	BGLCN10	B-site: 10% Ni
$Ba_{0.5}Gd_{0.8}La_{0.7}Co_{1.4}Fe_{0.4}Ni_{0.2}O_{6-\delta}$	BGLCFN2010	B-site: 20% Fe and 10% Ni
$Ba_{0.5}Gd_{0.8}La_{0.7}Co_{1.4}Fe_{0.2}Ni_{0.4}O_{6-\delta}$	BGLCFN1020	B-site: 10% Fe and 20% Ni
$Ba_{0.5}Gd_{0.8}La_{0.7}Co_{1.0}Fe_{0.6}Ni_{0.4}O_{6-\delta}$	BGLCFN3020	B-site: 30% Fe and 20% Ni
$Ba_{0.5}Gd_{0.8}La_{0.7}Co_{1.0}Fe_{0.2}Ni_{0.8}O_{6-\delta}$	BGLCFN1040	B-site: 10% Fe and 40% Ni
$Ba_{0.5}Gd_{0.8}La_{0.7}Co_{1.0}Ni_{1.0}O_{6-\delta}$	BGLCN50	B-site: 50% Ni

Table S2: Mean molar fractions (χ_i) of the elements in B-site substituted BGLC587. The values were acquired with EDS from three different areas of the catalyst powders. The errors are statistical and based on the three individual measurements. Values in red are the nominal molar fractions.

**Electrocatalyst**	A-site			B-site		
	χ_{Ba}	χ_{Gd}	χ_{La}	χ_{Co}	χ_{Fe}	χ_{Ni}
BGLC587	0.51 ± 0.01<br>0.5	0.71 ± 0.04<br>0.8	0.73 ± 0.02<br>0.7	2.05 ± 0.02<br>2.0	-	-
BGLCF10	0.51 ± 0.5<br>0.5	0.72 ± 0.06<br>0.8	0.73 ± 0.05<br>0.7	1.85 ± 0.04<br>1.8	0.19 ± 0.01<br>0.2	-
BGLCF30	0.5 ± 0.1<br>0.5	0.73 ± 0.09<br>0.8	0.68 ± 0.09<br>0.7	1.5 ± 0.1<br>1.4	0.57 ± 0.08<br>0.6	-
BGLCF50	0.50 ± 0.02<br>0.5	0.73 ± 0.01<br>0.8	0.70 ± 0.01<br>0.7	1.06 ± 0.02<br>1.0	1.02 ± 0.02<br>1.0	-
BGLCF70	0.59 ± 0.06<br>0.5	0.77 ± 0.04<br>0.8	0.71 ± 0.04<br>0.7	0.65 ± 0.03<br>0.6	1.38 ± 0.03<br>1.4	-
BGLF	0.52 ± 0.01<br>0.5	0.78 ± 0.01<br>0.8	0.72 ± 0.02<br>0.7	-	1.99 ± 0.02<br>2.0	-
BGLCFN2010	0.48 ± 0.08<br>0.5	0.73 ± 0.02<br>0.8	0.7 ± 0.1<br>0.7	1.5 ± 0.09<br>1.4	0.41 ± 0.02<br>0.4	0.21 ± 0.04<br>0.2
BGLCFN3020	0.53 ± 0.01<br>0.5	0.75 ± 0.02<br>0.8	0.76 ± 0.06<br>0.7	1.04 ± 0.02<br>1.0	0.60 ± 0.03<br>0.6	0.32 ± 0.03<br>0.4
BGLCN50	0.51 ± 0.01<br>0.5	0.8 ± 0.1<br>0.8	0.76 ± 0.01<br>0.7	1.08 ± 0.06<br>1.0	-	0.88 ± 0.2<br>1.0

Table S3: The double layer capacitances in the non-faradaic (capacitive) region as found by low and high scan rate CV data and EIS, in the onset potential region and at 10 mA cm^{-2}_{geo}. The rightmost column gives the BET surface area of the ECs.

Electrocatalyst	Non-Faradic region			OER onset	10 mA cm^{-2}_{geo}	Ex situ
	$C_{dl,CV,low}$ (mF)	$C_{dl,CV,high}$ (mF)	$C_{dl,EIS}$ (mF)	$C_{dl,EIS}$ (mF)	$C_{dl,EIS}$ (mF)	BET (m^2 g^{-1})
BGLC587	59	33	20	487	18	0.785
BGLCF10	48	30	40	94	18	0.823
BGLCF30	38	27	46	352	10	1.171
BGLCF50	51	34	44	90	17	1.604
BGLCF70	45	36	41	238	8	1.901
BGLCFN1020	33	19	51	55	17	0.827
BGLCFN1040	37	22	86	52	17	0.716
BGLCFN2010	42	27	46	175	19	0.605
BGLCFN3020	32	22	57	56	24	0.723
BGLCN10	39	25	64	69	23	0.541
BGLCN50	49	30	54	290	13	1.131
BGLF	43	23	36	176	24	3.552

Supplementary note 1

CV measurements were done in the non-faradaic region from 1.06 to 1.16 V vs RHE with scan rates varying from 5 to 200 mV s^{-1}. A representative figure of such measurements for BGLC587 is given in Figure S13a. The electrochemical surface area (ECSA) through the double layer capacitance, C_{dl} (F), was found by Equation S1,

$$I_c = \frac{I_{anodic} - I_{cathodic}}{2} \qquad \text{S1}$$

where I_c, is the charging current and I_{anodic} and $I_{cathodic}$ are currents associated with the positive and negative charging of the electrocatalyst surface respectively. C_{dl} was then found from the slope of Equation S2,

$$I_c = C_{dl} \cdot \nu \qquad \text{S2}$$

where it can be seen that C_{dl} has units $\mathrm{F} = \frac{\mathrm{A \cdot s}}{\mathrm{V}} = \frac{\mathrm{C}}{\mathrm{V}}$.

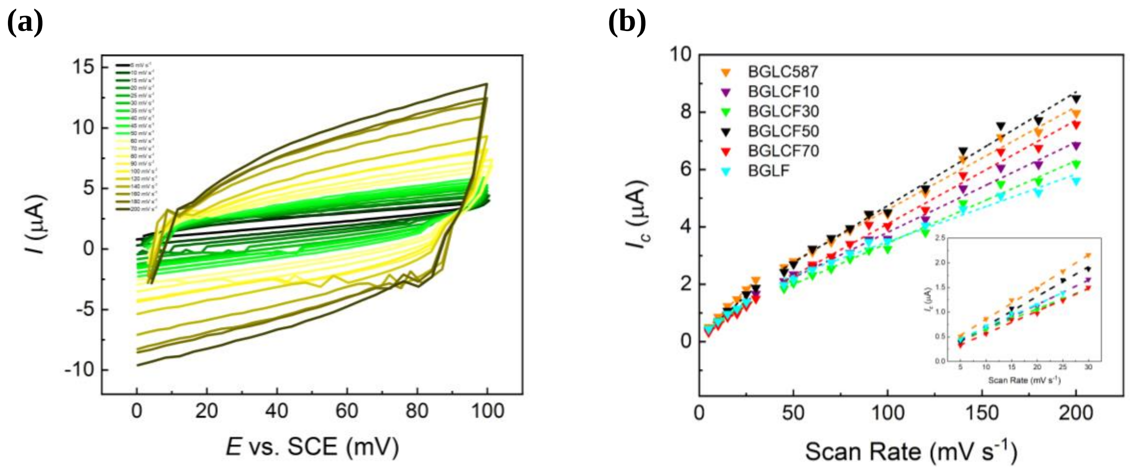

Figure S13: **(a)** CV curves in the non-faradaic region at scan rates 5-200 mV s^{-1} for BGLC587. **(b)** Plot of charging current vs scan rate for the Fe-substituted BGLC587. Two linear regions can be seen. The inset is a magnification of the data from the lower region.

Supplementary note 2

Electrochemical impedance spectroscopy (EIS) was performed at DC voltages corresponding to the non-faradaic region, the onset and a current density of 10 mA cm^{-2}. The EIS spectra were acquired with an AC frequency range of 100 kHz to 0.1 Hz and an AC amplitude of 10 mV rms. Zview (Scribner) was used to fit the data to the extended Randles circuit shown in Figure S13. The circuit consists of a solution resistance (R_s), which accounts for the electrolyte resistance as well as wires and connections. The value of R_s obtained in the non-faradaic potential region was used for iR-compensation of subsequent voltammetry curves.

Following R_s there are two R||C elements in series consisting of a resistor and constant phase element (CPE) in parallel with each other. The first R||C element is related to the resistive (R_d) and capacitive (C_d) behaviour as a result of diffusion by reactants close to the surface,[1, 2] while the second originates from the interfacial resistive (R_{int}) and capacitive (C_{int}) behaviour of the catalyst material. The constant phase element is used instead of a pure capacitor to account for the non-ideal capacitive behaviours in the system.

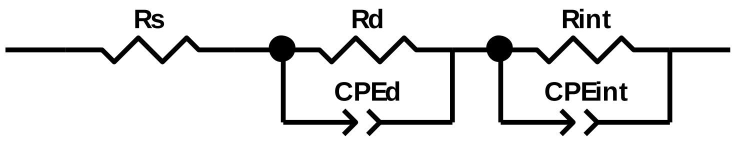

Figure S14: Extended Randles circuit used to fit the EIS spectra and extract the relevant resistances and capacitances.

The charge transfer resistance (R_{ct}) and the double layer capacitance (C_{dl}) were found from equations S3 and S4 described below, where we assume that both R||C elements contribute to these values. Nevertheless, the R||C element related to diffusion should give a rather small contribution as the rotating disk electrode minimizes the effect of mass transfer resistance.

$$R_{ct} = R_d + R_{int} \tag{S3}$$

$$C_{dl} = \frac{1}{\frac{1}{C_d} + \frac{1}{C_{int}}} \tag{S4}$$

Supplementary note 3

To obtain as close to steady-state conditions as possible for Tafel analysis, we performed stepped chronopotentiometry (CP) at current densities corresponding to the Tafel region of our catalysts. The voltage was measured for 5 min at 8 different current densities. The steady-state overpotentials were then found by calculating the mean potential from the last 30 s of each step. Figure S15a shows two typical stepped CP curves for BGLC587 and BGLCF70. Figure S15b compares the Tafel curves derived by stepped CP and LSV, with the latter obtained at 10 mV s^{-1} scan rate. It is clearly visible that the LSV derived Tafel region has a substantial contribution from capacitive current compared to the CP derived Tafel region. As we see, this impacts the value of the Tafel slope, but more importantly also the current density interval where it is linear, which should ideally span two to three decades.[3] Therefore, the CP method is more reliable, but both methods can be used to observe trends in Tafel slopes of a series of catalyst materials.

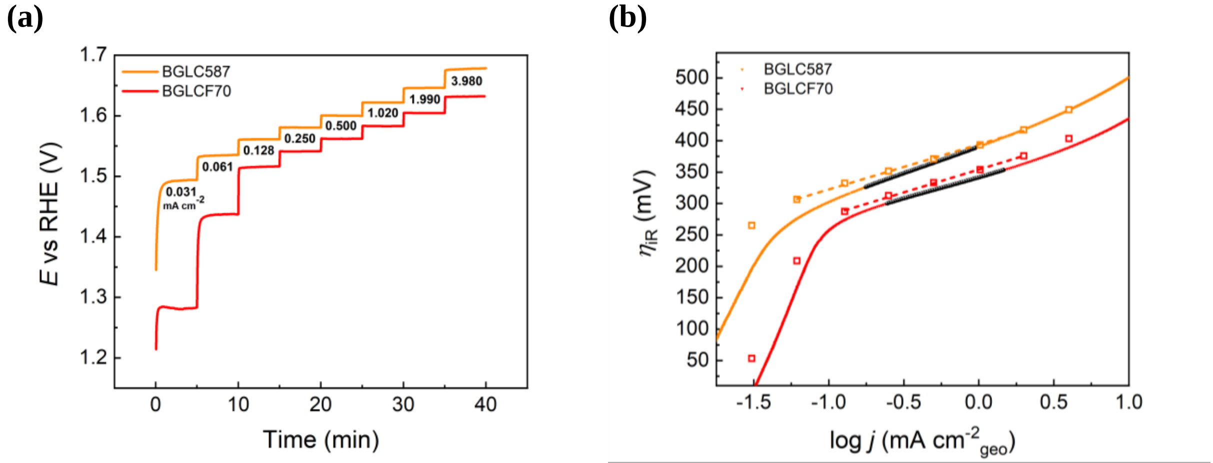

Figure S15: **(a)** Stepped CP curves of BGLC587 and BGLCF70 acquired at current densities corresponding to the potential region of the Tafel slopes. **(b)** Tafel curves from stepped CP in **(a)** (open squares) and LSV recorded with a scan rate of 10 mV s^{-1} (solid lines).

Supplementary note 4

The calibration curves of the saturated calomel electrode (SCE) reference electrode in different KOH concentrations are given in Figure S11a. From the intercepts with the potential axis, the E_{offset} values were found and plotted as a function of KOH concentration in Figure S11b. The blue dashed line in Figure S11b is the theoretical E_{offset} values as determined by Equation (1) in the main text, that gives the pH dependency of the potential vs the reversible hydrogen electrode (RHE). From the slope of the fitted line (red dashed line) in Figure S11b, it is observed that the trend found with the calibration method is in good agreement with the Nernstian behaviour of the theoretical line (blue line). In 1 M KOH, it was found that the experimental E_{offset} was -1057 mV, which is only 11 mV from the theoretical value of -1068 mV. This is not a significant difference and indicates that we were not too far from a theoretical system with electrolyte pH = 14.

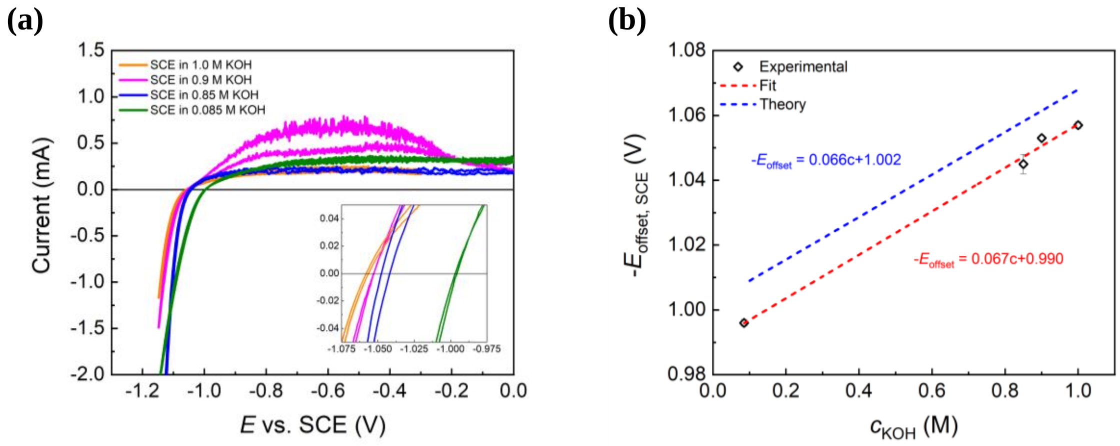

Figure S16: **(a)** Measured calibration curves for the SCE as function of KOH concentration. The intercepts with the x-axis of **(a)** give the E_{offset} values plotted in **(b)** which are compared to the theoretical values.

Supplementary note 5

C-paper electrodes (Alfa Aesar – same grade as Toray fuel cell paper) with a loading of 5 mg cm^{-2} were made by drop casting of the catalyst ink. The C-paper electrodes had a nominal surface area of 1 cm^2, where the middle part of the C-paper electrode was covered in Teflon™ tape, while the end was wrapped in aluminium foil to ensure a good, rigid contact. A representative photo of a C-paper electrode loaded with catalyst is given in Figure S17b. The C-paper electrodes were cleaned and conditioned following the same procedure as for the RDE before CP measurements.

(a) **(b)**

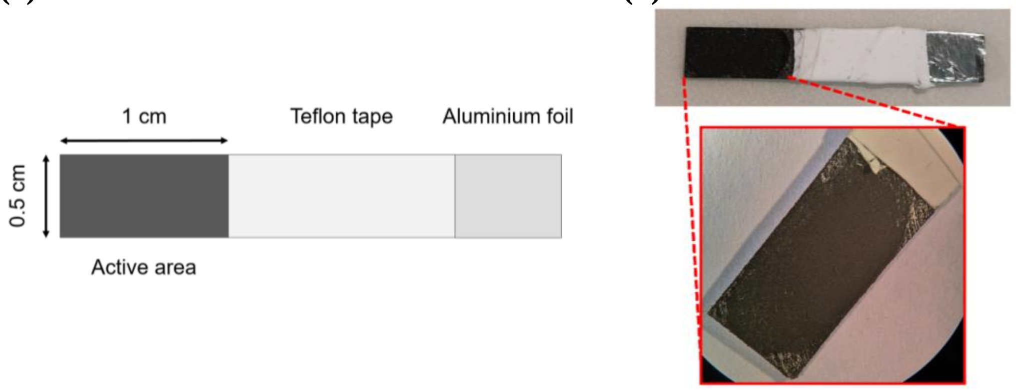

Figure S17: **(a)** C-paper electrode schematic. **(b)** Photo of an actual C-paper electrode with 5 mg cm^{-2} loading. The zoomed in photo is representative of the 1 cm^2 electrode area after catalyst loading.

References

1. Zhu, J., et al., *Double Perovskite Cobaltites Integrated in a Monolithic and Noble Metal-Free Photoelectrochemical Device for Efficient Water Splitting.* ACS Applied Materials & Interfaces, 2021. **13**(17): p. 20313-20325.
2. Bredar, A.R., et al., *Electrochemical impedance spectroscopy of metal oxide electrodes for energy applications.* ACS Applied Energy Materials, 2020. **3**(1): p. 66-98.
3. Anantharaj, S., et al., *Precision and correctness in the evaluation of electrocatalytic water splitting: revisiting activity parameters with a critical assessment.* Energy & Environmental Science, 2018. **11**(4): p. 744-771.
